# Operating pesticide use reduction within the boundary of food security in peri-urban settings

**DOI:** 10.1016/j.fmre.2022.04.003

**Published:** 2022-04-18

**Authors:** Yuquan W. Zhang, Bruce A. McCarl, Zhengwei Cao, Qiang Li, Shaohua Yang, Huiwen Liu

**Affiliations:** a China-UK Low Carbon College, Shanghai Jiao Tong University, No. 3 Yinlian Road, Pudong New Area, Shanghai 201306, China; b Department of Agricultural Economics, Texas A&M University, 2124 TAMU, College Station, Texas 77843, USA; c School of Agriculture and Biology, Shanghai Jiao Tong University, No. 800 Dongchuan Road, Minhang District, Shanghai 200240, China

**Keywords:** Pesticide use reduction, Shanghai Agricultural Sector Model (SH-ASM), Peri-urban agriculture, Crop budget, Crop mix

## Abstract

Pesticide use in peri-urban areas affects the urban environment and public health, and reducing the use may present food security issues for urban dwellers. In this study, we explore how a municipality-adopted goal of a 20% reduction in pesticide use could be achieved, along with local food security and environmental implications, for Shanghai located in the densely populated East China. A regional Shanghai Agricultural Sector Model incorporating district- and technology-varying crop budgets, was developed to simulate the effects of pesticide reduction policy. Here we find that achieving the reduction goal had the largest implications in districts with high pesticide use totals and intensities, potentially reducing pesticide non-point source pollution in the Yangtze River Estuary and Dianshan Lake; the production levels of rice and leafy vegetables would be most affected; and adopting machinery that allows more precise pesticide application modulates these results. Moreover, imposing the requirements at the district-level caused more severe local food security concerns, and less environmental benefits. Furthermore, a closed Shanghai's agricultural economy would substantially enlarge the regional heterogeneity in the above-mentioned outcomes. Exploring the effects of a quantity control policy on current-use pesticides at different aggregation levels has important implications for regulating the use of agrochemicals.

## Highlights

• Pesticide use threatens environmental and human health, especially near urban areas
• Shanghai has set pesticide use reduction goals
• A new optimization model was developed to simulate Shanghai's peri-urban agricultural sector
• Regional reduction mostly affected agricultural areas with intense pesticide use
• Pesticide pollution reduction is expected in Yangtze River Estuary and Dianshan Lake

## Graphical abstract

**Figure:**
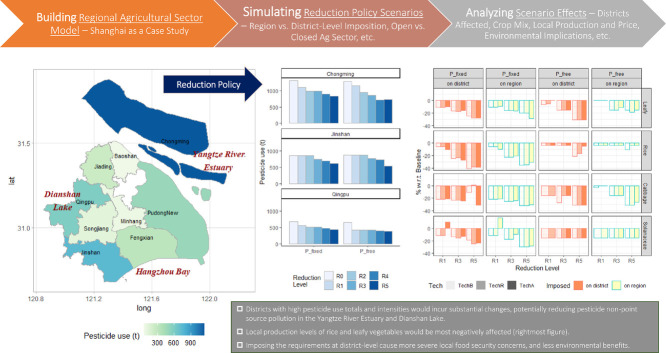

## 1 Introduction

Appropriate pesticide use in farming has received increasing, widespread global attention since the late 1950s, during which Stern et al. [1] initially proposed the concept of integrated control and then *Silent Spring* authored by Carson [2] got published in 1962. European countries and the United States (US) began addressing concerns over the harmful aspects of pesticide use in the 1970s by promoting Integrated Pest Management (IPM) [3]. Increasing pesticide use, and sometimes overuse, threatens environmental and human health [2,4], contributes to air [5], soil and water pollution [4,6] and biodiversity loss [7], and raises food safety and security challenges [8,9]. In response to these considerations, stringent regulations over pesticide use had emerged in recent decades [10], e.g., France adopted a 10-year Ecophyto plan in 2008, aiming to halve its agricultural and non-agricultural pesticide use [11]. Regulatory controls over pesticides had also become remarkably tightening since the mid-2010s in major agricultural production developing countries, including Brazil, China, India, Kenya, etc [12].

Consulting on the Pesticide Environmental Accounting (PEA) system, the externalities of pesticide use mainly involve three aspects: 1) occupational health that concerns the safety of applicators and pickers; 2) consumer health that is at risk due to pesticide residues left on food and water pollution caused by pesticide leaching and runoff; and 3) ecological health that concerns the aquatic and terrestrial life such as birds, bees, and beneficial insects [13]. Environmental and health damage due to pesticides is more severe for agricultural activities in peri-urban areas than in rural regions [14,15]. There are widespread recorded safety and health concerns regarding pesticide residues in food, particularly vegetables [8]. Note that for peri-urban agriculture, the share of horticulture crops can be close to that of field crops, and may be substantially larger than under the conventional rural settings [16]. This deserves particular attention because research showed that human toxicity and aquatic ecotoxicity hazards associated with horticulture crops are 5 to 10 times more than field crops, due to high levels of agrochemicals usage [17]. Reducing pesticide use can then improve urban public and environmental health, as the urban population is dense and exposed to peri-urban pesticide application and runoff. What compounds the issue is that farmers located closer to cities often use more pesticides, due to their larger financial resources and higher hired labor market wage rates [18]. Moreover, urbanization and industrialization are identified as the principal factors reducing productive croplands, particularly in mega-urban regions of Asia and Africa [19,20], which in turn threatens local food security. Additionally, although land availability is a limiting factor for Shanghai's agricultural production, the residents’ preferences over leafy greens has arguably mandated a self-sufficient rate of leafy greens at around 85% [21]. Therefore, it is crucial to examine policies bearing agri-environmental significance and food security implications in the peri-urban settings.

Pesticide use is being reduced in China, and particularly the metropolitan Shanghai area with 24.87+ million residents. In 2015, the central government of China launched a “2020 Action Plan” that called for no increase in agricultural pesticide use by 2020 [22]. Within China, the Shanghai Municipality strengthened the national plan for its peri-urban environment by setting a reduction goal of 20% relative to the usage in 2015 [23]. Since these announcements, the pesticide use in Shanghai substantially decreased from an overall 4,415 t in 2015 to 2,771 t in 2019, which is a decrease of 37.24%. Meanwhile, the total pesticide use in China incurred a reduction of 21.94%, from 1,782,969 t in 2015 to 1,391,747 t in 2019 [24,25].

Despite the above average achievement in reducing pesticide use, significant decreases in agricultural land use and crop production have accompanied this reduction. The planted area in Shanghai shrank from 351,700 ha in 2015 to 285,300 ha in 2018, which is a decrease of 18.88% [26]. The production of staple crops decreased by 14.29% from 1,120,000 t in 2015 to 960,000 t in 2019, and vegetable production decreased by 26.37% from 3,640,000 t in 2015 to 2,680,000 t in 2019 [24,25]. Although these numbers suggest some increases in agricultural productivity per unit of cropland in the Shanghai Region, the agrochemicals use reduction policies might have contributed to the compromised local food security and reduced agricultural land use, amidst the backdrop of increasing urbanization. This is against the implicit expectation that local food security should have been guaranteed.

There have been studies examining if controlling pesticide use would be in conflict with crop production and economic returns, e.g., based on examining farm-level data during the period of 2009-2011 across France, Lechenet et al. [27] suggested it is possible to reduce pesticide use without much affecting crop productivity and farm profit. However, using 2009-2013 data on national pesticide sales, treatment frequency index (TFI), and water pollution, Hossard et al. [11] found the use reduction targets were far from achieved. Despite of the mixed results for France, a case study of Denmark showed success in reducing the pesticide load [10], proposing the use of risk-based indicators involving human health, ecotoxicity, and environmental fate to help mitigate the pesticide risks. For developing countries, it would be challenging to adopt a monitoring system that records spatially explicit data on PL (pesticide load) as in Denmark [10], due to a variety of limiting conditions. More straightforward quantitative indicators such as usage per unit of land (kg/ha) and total usage levels may prevail instead as metrics of policy goals.

Upon reviewing pesticide reduction policies with a focus in Europe, Möhring et al. [28] suggested it is crucial to factor in the intensive, extensive, and super-extensive margins for policy-making, and the target be designed to accommodate the regional and/or landscape level situations. In other words, the human responses in the forms of changing crop mix and technology use, be they exercised individually or collectively, may impose substantial effects on materializing the intended outcome of pesticide policies. Additionally, the policy outcomes can diverge considerably with regard to the aggregation level. A case study in the United Kingdom (UK) demonstrated the value of the systems-based approach for a surface water catchment relative to mere adoption of technical means, despite that there existed barriers preventing joint efforts between farmers to mitigate pesticide pollution [29]. A recent US study that compared farm-level budgeting and sectoral simulation approaches suggested that, upon introduction of notable changes in regulating pesticide use, simple farm-level budgeting may result in substantial bias in estimating welfare effects on producers [30]; thus, modeling with a broader scope is required to better analyze and design pesticide policies.

Turning to Shanghai, owing to limited public information regarding the aforementioned decreases in crop production and pesticide use, and to determine if local production needed to decrease drastically, this study aimed to explore how the 2015 regional plan would have affected the peri-urban agricultural sector of Shanghai. Specifically, (1) Regarding the intensive and extensive margins, how would pesticide use reduction efforts vary by crop and district (sub-region) in addition to technology use, and what are the accompanying environmental implications? (2) How would the 2015 reduction policy affect local agricultural production and prices, the two fundamental dimensions of food security relating to physical availability and affordability [31]? (3) Would the finer scale imposition at district (sub-region) level bring further difference? (4) Though Shanghai's agricultural sector is well integrated with the rest of China [32,33], what if it were a closed economy wherein emphases were attached to local produce?

We conducted a scenario-based analysis to address these questions, by developing and using a new regional agricultural sector model, Shanghai Agricultural Sector Model (SH-ASM, see Methods 2.1) for the Shanghai peri-urban area that simulates farmer representative decisions. Combined with data from major statistics yearbooks on agriculture for Shanghai and a 2020 survey consisting of 532 usable samples across suburban Shanghai (see Methods 2.2), the model solves for crop mix, pesticide use, market equilibriums, etc., and derives environmental implications, under various scenarios concerning the effects of overall reduction level, technology use, district-level imposition of use reduction, and closedness of local agricultural sector (see Methods 2.3). The potential contribution of this study is at least twofold: first, it delved into previously unexplored quantity control possibilities for pesticide use policies, deriving systems-based insights that potentially enable pesticide use reduction via the common market mechanism; second, it enriched the global database of case studies dedicated to urban and peri-urban agriculture, integrating the dimensions of both food security and agri-environmental aspects concerning pesticide use in particular.

## 2 Methods and data

### 2.1 Simulation of Shanghai's peri-urban agricultural sector

To explore the implications of pesticide usage reductions for the Shanghai region, a model integrating crop mix and management shifts with market reactions is required. Here we employed a framework that simulated profit-maximizing farmer behaviors that reallocate production activities or crop enterprises, along with cost-minimizing consumer demand for multiple agricultural commodities. Thus, a regional Shanghai Agricultural Sector Model (SH-ASM) written in the General Algebraic Modeling System (GAMS) programming language was developed [34].

The mathematical structure was based on that used in the USA Forest and Agricultural Sector Optimization Model (FASOM) model [35] and the theory explained by McCarl and Spreen [36] and then evolved as revisited by McCarl et al. [37]. The model represents profit-maximizing land allocation between crop enterprises considering prices, yields, input usage, production costs, and pesticide use, among other factors, along with cost-minimizing consumer choices.

The optimization is subject to the system of constraints shown below.

(1) $$\max\;\sum_{m}\int_{0}^{Y_{m}}P_{dm}\left(Y_{m}\right)dY_{m}-\sum_{i}\int_{0}^{X_{i}}P_{si}\left(X_{i}\right)dX_{i}$$

(2) $$s.t.\;\;Y_{m}-\sum_{n}\sum_{k}c_{mnk}Q_{nk}\leq 0,\;\forall m$$

(3) $$-X_{i}+\sum_{n}\sum_{k}a_{ink}Q_{nk}\leq 0,\;\forall i$$

(4) $$\sum_{k}b_{jnk}Q_{nk}\leq Z_{jn},\;\forall j,n$$

(5) $$Y_{m},X_{i},Q_{nk}\geq 0,\forall m,i,n,k$$

where m is the agricultural commodity, i denotes the purchased inputs, j represents the regionally fixed resources, n denotes the district, and k is the production process.

Eq. 1 maximizes the sum of the consumer and producer surpluses within the Shanghai peri-urban agricultural sector, encompassing the consumption of agricultural commodities $Y_{m}$ and supply of inputs $X_{i}$.

Eq. 2 balances output sales $Y_{m}$ with the yield of production processes $Q_{nk}$, where $c_{mnk}$ is the per-unit yield.

Eq. 3 links input purchases $X_{i}$ with use by the production processes $Q_{nk}$, where $a_{ink}$ is the per-unit input usage.

Eq. 4 limits the resource availability, where $b_{jnk}$ represents the resource usage per unit of production, and $Z_{jn}$ is the resources endowment.

Finally, Eq. 5 states that the output $Y_{m}$, input $X_{i}$, and production process $Q_{nk}$ levels must be non-negative.

The GAMS code [34] for the core model structure is provided in Supplemental File S1.

Compared with other existing well-established agricultural sector models, the SH-ASM differs in three main aspects: 1) Other models are usually at national or world level (e.g. CASM [38], CAPSiM [39], and GLOBIOM [40]), incorporating sub-national or national level production activities. It is challenging to apply such models to develop detailed insights into peri-urban agriculture, such as the case of Shanghai that covers 9 suburban districts. While there are certainly regional models such as SWAP [41] and EDSIM [42], they were primarily used for water use management studies. 2) Other models often treat “vegetables and fruits” aggregately, whereas among the 15 crops simulated by the SH-ASM, 80% are non-grain crops. For instance, leafy greens (mainly consisting of Pak Choi (*Brassica chinensis*)), Solanaceae (tomato (*Solanum lycopersicum*)), beans (*Phaseolus*), strawberry (*Fragaria ananassa*), etc. 3) While other models may include data on use of chemicals (e.g., nitrogen fertilizer), they usually lack explicit data on pesticide use. Herein the SH-ASM database includes crop- and district-specific inputs usage including pesticides.

### 2.2 Sector model data

**Fig. 1:**
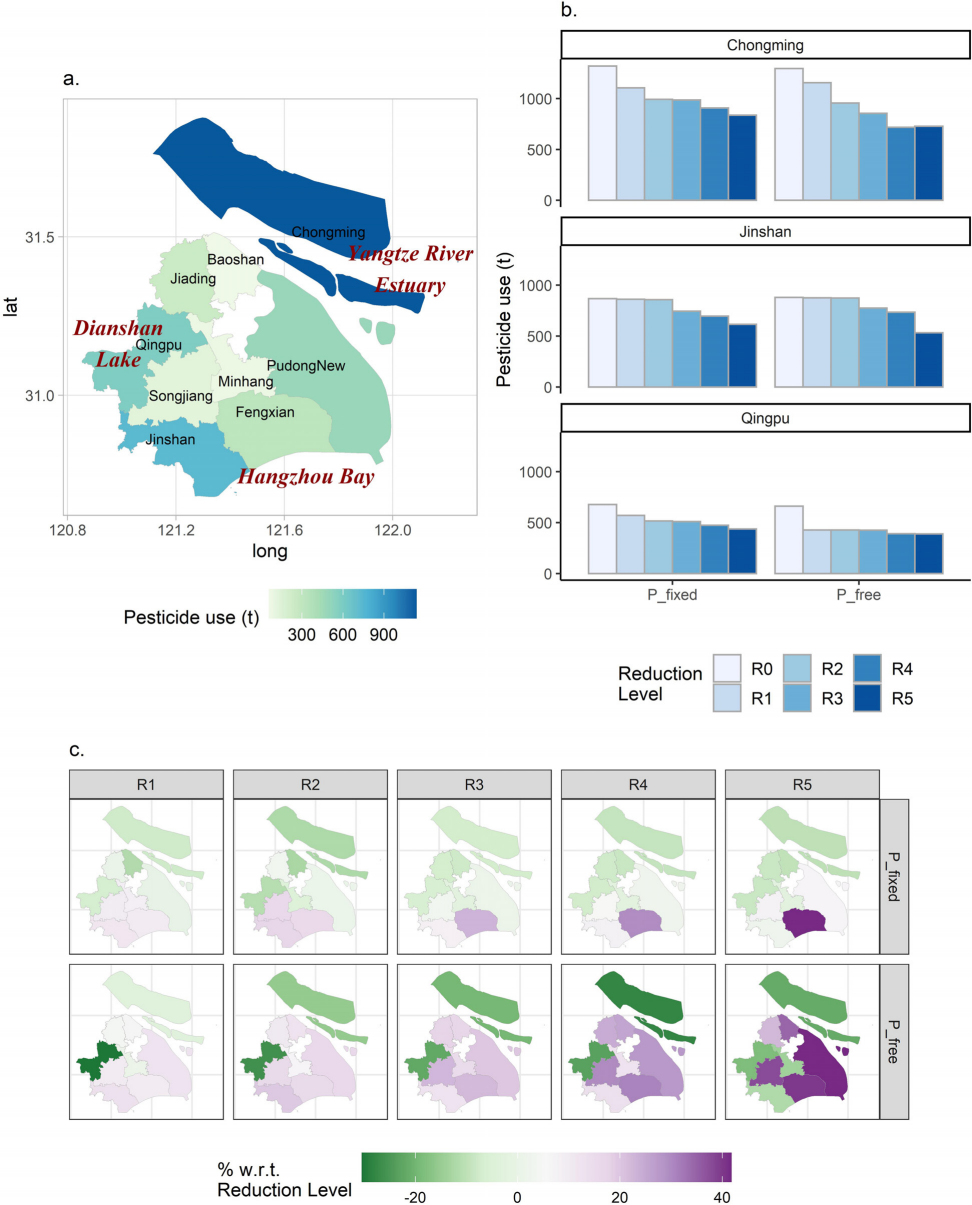
**District-level totals of or changes in pesticide use across peri-urban Shanghai under different scenarios.** (a) Base district-level totals of pesticide use across peri-urban Shanghai, 2015. Low-mid-high color gradient is used to indicate the differences. Sitting at the Yangtze River Estuary, Chongming exhibits the highest level of use; Qingpu, bordering the Dianshan Lake that provides drinking water sources, shows a mid-level; Jinshan and Fengxian, facing the Hangzhou Bay, show high and low levels of use, respectively. (b) Projected pesticide use levels for the top 3 districts (Chongming, Jinshan, and Qingpu) under various “TechB + Regionwide imposition” scenarios (see Methods Table 2) assuming zero adoption of chemicals application machinery and no prescribed district-level reduction goals across Shanghai. “P_fixed” refers to an open agricultural economy, under which Shanghai acts as a price taker, while “P_free” indicates a closed agricultural economy, assuming no perfect substitutes exist for local produce. Color gradient is used to exhibit low-highs under different reduction levels (R0-R5). (c) Projected percentage changes in pesticide use levels with respect to assumed district-level usage under different reduction level scenarios (R1-R5), for each district across peri-urban Shanghai under varying “TechB + Regionwide imposition” scenarios. Diverging color gradient indicates “less or more than” the assumed district-level usages. Compared with “P_fixed”, the projected percentage changes were more pronounced under the “P_free” scenarios which put greater emphases on local produce. A full version of (c) can be found in Supplemental File S3 (Fig. S1).

As mentioned above, the SH-ASM covers nine districts and fifteen crops. Fig. 1a (in Results 3.1) provides a geographical overview of the nine suburban districts modeled in the Shanghai study. The data [34] used for modeling were obtained from several sources. The baseline crop budgets by district were derived from the *Shanghai Countryside Statistical Yearbook* 2016 [43]. Data regarding the historical crop mix were obtained from 2015 district-level data (*Shanghai Countryside Statistical Yearbook* 2016) and historical data on staple and cash crops [44]. Data regarding market prices and quantities were obtained from the Shanghai Municipal Commission of Agriculture and Rural Affairs, and the own-price elasticities were obtained from the literature [45,46]. To obtain district- and crop-specific inputs usage and to estimate the effects of pesticide-saving technologies on rice (*Oryza sativa*) production and cost, we used data collected via a questionnaire, the details of which are provided in Supplemental File S2 (hereafter called Survey 2020). We then computed differences in the yield, cost, and pesticide use with and without chemicals application machinery, and the percentage differences between the statistical averages of the two cases were then used to construct technology use-specific crop budgets (Table 1). The machinery purchase cost was annualized following the equivalent annual cost (EAC) method [47] with “10 years” as the machine life-span and 5% as the discount rate.

**Table 1:** **Citywide Shanghai rice budget derived based on Survey 2020**.

Crop Budget Item	Sample	Not Using Machinery for Application of Chemicals	Mechanized Application of Chemicals
	(n=532)	(n=251)	(n=281)
Yield (kg/ha)	8064.94	7943.18	8085.23
Fertilizer (kg/ha)	997.40	1009.09	995.45
Pesticides (kg/ha)	18.00	18.75	17.87
Labor Hired Price (RMB yuan/person month)	1915.58	1863.64	1924.24
Labor (Family and Hired, person month/ha)	13.58	14.83	13.37
Machinery Cost, Annualized (RMB yuan/ha)	4489.26	3942.43	4580.40
Machinery Purchase Cost, Annualized (RMB yuan/ha)	2200.30	1794.70	2267.90
Machinery Rent (RMB yuan/ha)	2288.96	2147.73	2312.50

Notes: Overall, 540 farmer responses were collected for the survey, covering all nine suburban districts of Shanghai, with each district being assigned 60 responses. After removing the invalid samples, 532 remained, 251 of which reported that they did not use machinery for chemical application, and 281 reported that they did, whether the machinery was self-purchased or rented. The mean values of the sample (subsamples) are used for the items reported above.

The Survey 2020 was performed via contracting a professional local survey company (www.wenjuan.com) during February and March, 2020. Due to the then outbreak of COVID-19, the survey was done electronically. Snowball sampling was involved to find suitable responders: for each district, the survey company firstly asked the contacts with agri-business tags in their database, and then the contacts referred to farmers they knew to participate in the survey. To prevent duplication, one-time use links were sent to the participating farmers, and the uniqueness of the IP address associated with the participant's smartphone or computer was checked to guarantee the data integrity. The purpose of the survey, data anonymity, and the use were introduced to the participants firstly, then they could move on to fill in the form as they wish. They could also exit the survey anytime they wish. Informed consent was thus substantiated and obtained. The overall collected data were then examined from multiple aspects such as farm size, phases of machinery use, inputs usage, years of practice, age, gender of household decision-maker, etc. to ensure the quality.

### 2.3 Analyzed scenario

A set of pesticide use "quantity control” and mechanized application scenarios were analyzed, as shown in Table 2.

**Table 2:** **Scenarios considered in this study**.

Dimension 1: Pesticide use reduction levels		
	Level	Description
	R0	0% reduction in total regional pesticide use
	R1	to achieve 10% reduction in total regional pesticide use
	R2	to achieve 15% reduction in total regional pesticide use
	R3	to achieve 20% reduction, as mentioned in the 2015 plan by the Shanghai Municipality [23]
	R4	to achieve 25% reduction in total regional pesticide use
	R5	to achieve 30% reduction in total regional pesticide use
Dimension 2: Adoption of chemicals application machinery		
	Technology	Description
	TechB	Chemicals application machinery is not adopted
	TechR	Chemicals application machinery is only available for rice (*Oryza sativa*) production; this causes rice yields and machinery costs to increase, while pesticide use and labor costs decrease
	TechA	Chemicals application machinery is available for all crops, and the corresponding crop budgets were modified based on rice data
Dimension 3: Imposition		
	Level	Description
	on district	Pesticide use reduction rates applied onto each district in peri-urban Shanghai
	on region	Pesticide use reduction rates applied onto the entire peri-urban Shanghai
Dimension 4: Open or closed regional agricultural economy		
	Case	Description
	P_fixed	Shanghai's agricultural sector is well connected to the rest of China, acting as a price taker
	P_free	Assume Shanghai is having a closed regional agricultural economy, which puts emphasis on local produce. Note that this is not to say Shanghai does not import from or export to other areas, but rather there are no external good substitutes for local produce

Regionwide reduction levels ranging from 10% to 30%, along with a base scenario of zero reduction, were applied in this study. Also, three alternatives were introduced for the adoption of spraying machines that can increase the precision of pesticide application. Moreover, the district-level and the regionwide imposition of the pesticide use reduction rates were considered. Additionally, two extreme cases including the “fixed price” and the hypothetical “closed agricultural economy” were modeled. Thus, 66 alternative scenarios were simulated in this study. For scenario comparison, the Fisher price index [48] was used for analyzing the summary price effects.

The model validation results concerning the comparisons of the base scenario (R0 and TechB) outputs under the “P_fixed” status and observed data are presented in Table 3. The “P_free” status is displayed in Table 4. As shown in the tables, the ratios of the observed values to the modeled values were mostly close to 1.00, particularly for price and acreage. Regarding production, the ratios for all crops, excluding a few minor species, exceeded 0.85. These validation results suggest that the model generated a reasonable approximation of the real-world baseline for Shanghai's agricultural sector.

**Table 3:** **Validation results for the Shanghai agricultural sector model under the “P_fixed” status**.

Crop	Acreage (ha)			Production (ton)			Price (RMB yuan/kg)		
	Observed^a^	Model Baseline	Ratio	Observed^b^	Model Baseline	Ratio	Observed^c^	Model Baseline	Ratio
Rice	85656	87414	1.021	841000	753873	0.896	4.35	4.350	1.000
Wheat	38397	35598	0.927	199200	164737	0.827	2.76	2.760	1.000
Barley	7183	8009	1.115	45200	36160	0.800	2.55	2.717	1.065
Melon	6937	7545	1.088	264500	226542	0.856	5.52	5.520	1.000
Rapeseed	3873	3944	1.018	9600	8701	0.906	3.04	3.038	1.000
Strawberry	1276	1143	0.896	22100	19774	0.895	15.00	14.997	1.000
Fruit	20067	21161	1.055	326900	337969	1.034	9.28	9.276	1.000
Leafy	52125	50911	0.977	1404000	1339755	0.954	2.50	2.501	1.000
Pekinensis	3361	3118	0.928	163500	146749	0.898	1.41	1.410	1.000
Cabbage	11154	12998	1.165	550500	484307	0.880	1.43	1.430	1.000
Root	5587	5265	0.942	190700	174143	0.913	1.63	1.630	1.000
Cucurbit	5977	5669	0.948	244900	224788	0.918	4.16	4.161	1.000
Beans	10406	9485	0.911	226700	210744	0.930	6.12	6.118	1.000
Solanaceae	7912	7260	0.918	300900	268735	0.893	5.01	5.010	1.000
Allium	1964	2354	1.199	76500	70363	0.920	2.66	2.660	1.000
Average, arithmetic			1.007			0.901			1.004
Average, weighted by revenue^d^			1.003			0.928			1.000

Notes: a) crop acreage data were obtained from the *Shanghai Countryside Statistical Yearbook* 2016 [43]; b) production data were also obtained from the *Shanghai Countryside Statistical Yearbook* 2016 [43]; c) crop price data were obtained from the Shanghai Municipal Commission of Agriculture and Rural Affairs; d) for each crop, revenue is the product of observed production and observed price.

**Table 4:** **Validation results for the Shanghai agricultural sector model under the “P_free” status**.

Crop	Acreage (ha)			Production (ton)			Price (RMB yuan/kg)		
	Observed^a^	Model Baseline	Ratio	Observed^b^	Model Baseline	Ratio	Observed^c^	Model Baseline	Ratio
Rice	85656	83627	0.976	841000	719832	0.856	4.35	4.304	0.989
Wheat	38397	35158	0.916	199200	163344	0.820	2.76	3.225	1.168
Barley	7183	8209	1.143	45200	37064	0.820	2.55	2.834	1.111
Melon	6937	7264	1.047	264500	216890	0.820	5.52	5.444	0.986
Rapeseed	3873	4322	1.116	9600	9381	0.977	3.04	3.093	1.018
Strawberry	1276	1250	0.980	22100	21437	0.970	15.00	15.001	1.000
Fruit	20067	20198	1.007	326900	317093	0.970	9.28	8.977	0.967
Leafy	52125	52764	1.012	1404000	1361880	0.970	2.50	2.476	0.990
Pekinensis	3361	3283	0.977	163500	158595	0.970	1.41	1.461	1.036
Cabbage	11154	14255	1.278	550500	533985	0.970	1.43	1.493	1.044
Root	5587	5494	0.983	190700	184979	0.970	1.63	1.695	1.040
Cucurbit	5977	5959	0.997	244900	237553	0.970	4.16	4.192	1.008
Beans	10406	9552	0.918	226700	219899	0.970	6.12	6.168	1.008
Solanaceae	7912	8043	1.017	300900	291873	0.970	5.01	5.011	1.000
Allium	1964	2498	1.272	76500	74205	0.970	2.66	2.59	0.974
Average, arithmetic			1.043			0.933			1.023
Average, weighted by revenue^d^			1.010			0.929			0.999

Notes: a) crop acreage data were obtained from the *Shanghai Countryside Statistical Yearbook* 2016 [43]; b) production data were also obtained from the *Shanghai Countryside Statistical Yearbook* 2016 [43]; c) crop price data were obtained from the Shanghai Municipal Commission of Agriculture and Rural Affairs; d) for each crop, revenue is the product of observed production and observed price.

## 3 Results

### 3.1 Regional total pesticide usage and intensity

We first investigated how pesticide usage reductions were spread across the peri-urban districts (Fig. 1). Under the “regionwide imposition” scenarios that allow flexibility in cross-district reallocation of pesticide use, the reductions varied between districts, with more severe reductions occurring in districts with larger usage totals (Fig. 1a, b). In particular, as shown in Fig. 1c, Chongming (northern islands) and Qingpu (westernmost), the two major agricultural districts of Shanghai, committed deeper reductions leading to usage lower than the levels prescribed by the “imposed on district” dimension (Chongming: -12.09% to -7.53%; Qingpu: -10.87% to -6.39%, across R1-R5 reduction levels under P_fixed status).

On the other hand, Jinshan (southernmost), Fengxian (south coastal), and PudongNew (eastern) exhibited usage totals higher than the district target levels in most cases (Jinshan: +2.03% to +17.07%; Fengxian: +9.68% to +41.69%; PudongNew: -0.73% to +6.69%, across P_fixed R1-R5 reduction levels).

The results above suggest that to reduce pesticide use, it would be less sectoral welfare-decreasing if such reductions occur in Chongming and Qingpu. The former's economic output per unit of land is in general lower than other districts, due to lower yields. Hence, when the permitted amount of pesticide use becomes increasingly scarce, Chongming would be among the first to see a shrink in acreage. Regarding the latter, due to higher pesticide use intensity in general, the shadow costs of pesticide use cuts would cause a decrease in Qingpu's acreage.

Compared with the fixed price (P_fixed) case which assumed Shanghai's agricultural sector as a price taker, the closed agricultural economy case (P_free) demonstrated notably severe contrasts between the increasing and the decreasing districts. For example, Chongming saw -27.98% to -3.25% decreases, whereas PudongNew showed +12.22% to +41.73% increases, under varying P_free reduction levels.

One may note that the decreasing districts in the North and the West of Shanghai are in proximity to the Yangtze River Estuary and the Dianshan Lake, respectively, implying potential reductions in pesticide-related pollution load for these two water bodies. Meanwhile, the increasing districts in the South of Shanghai are facing the Hangzhou Bay, which may then need more efforts to mitigate pesticide pollution.

**Fig. 2:**
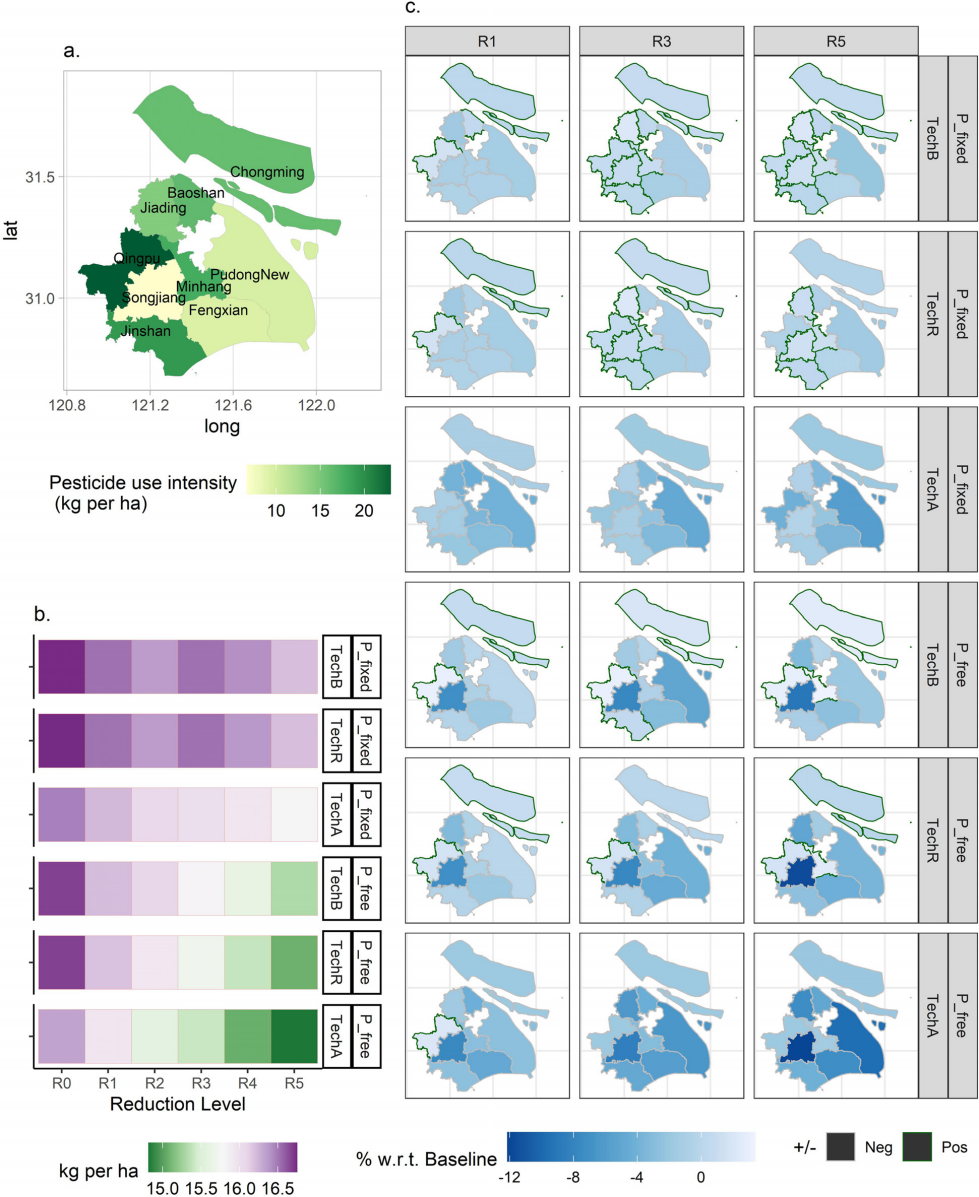
**Levels and changes in pesticide use intensity across Shanghai under various scenarios.** (a) Base pesticide use intensity (kg/ha) across suburban districts in Shanghai, 2015. Low-high color gradient is used. Qingpu (westernmost) tops the use intensity, while Songjiang (southwestern) shows the lowest level. (b) Projected use intensity at the Shanghai level under “Regionwide imposition” scenarios. Diverging color gradient is used. Effects of chemicals application machinery adoption appear notable, where TechR and TechA that allow the possibility of adoption for rice only and for all crops, respectively, exhibit lower intensities, with the “P_free” closed agricultural economy bringing about deeper cuts than the “P_fixed” price-taker conditions. As the reduction level gets deeper (10% under R0 to 30% under R5), pesticide use intensity decreases from 16.0+ to 15.0- kg/ha. (c) Projected percentage changes in use intensity across districts in Shanghai under various “Regionwide imposition” scenarios. To save space, R1, R3, and R5 are selected to demonstrate the changing patterns of use intensity changes. Darker colors indicate larger decreases. Districts with borders in dark green are seeing increases, albeit slight, in pesticide use intensity. With the assumptions of the “P_free” closed agricultural economy, more pronounced decreases would show across districts in Shanghai, especially Songjiang (southwestern) with the least baseline use total (Fig. 1a) and use intensity (Fig. 2a). A full version of panel (c) is provided in Supplemental File S3 (Fig. S2).

The spatially varying differences in the pesticide usage manifested in Fig. 2c indicate that the largest increases in intensity would occur in Qingpu (the westernmost district with comparatively high yields for many crops) and Chongming (the largest agricultural district), under the influence of regionwide imposition scenarios (Qingpu: +2.95% to +3.02%; Chongming: +0.73% to +2.66%, under TechB R1-R5 reduction levels). This suggests an underlying shift to or rise in the proportion of pesticide-intensive crop production in major agricultural districts. However, slight to moderate decreases in the pesticide use intensity were observed in Fengxian (south coastal; -3.11% to -1.89%) and PudongNew (eastern; -5.28% to -0.13%, under TechB R1-R5), suggesting a shift away from or a reduced share of pesticide-intensive crop production in these districts. In general, deeper reduction goals would widen the gaps between the low and high use intensity districts.

Under the TechR and TechA scenarios that allow the possibility of adopting chemicals application machinery (for rice only and for all crops, respectively), the districts seeing increases in use intensity previously now exhibited decreases, especially for Jinshan (the southern district facing the Hangzhou Bay) with a high base level (19.29 kg/ha, Fig. 2a). This also suggests that individual “intensity control” measures, such as adopting pesticide sprayer machinery, would impact the usage intensity more effectively than regional quantity control, where the individual refers to the district-level farming representative within the context of the SH-ASM.

Furthermore, both the widening effects of the reduction plan and the modulating effects of mechanizing chemicals application on the regional disparity of use intensities, would become more apparent under the P_free scenarios, which implicitly required more of the adaptation to take place within peri-urban Shanghai.

### 3.2 Crop mix movements

**Fig. 3:**
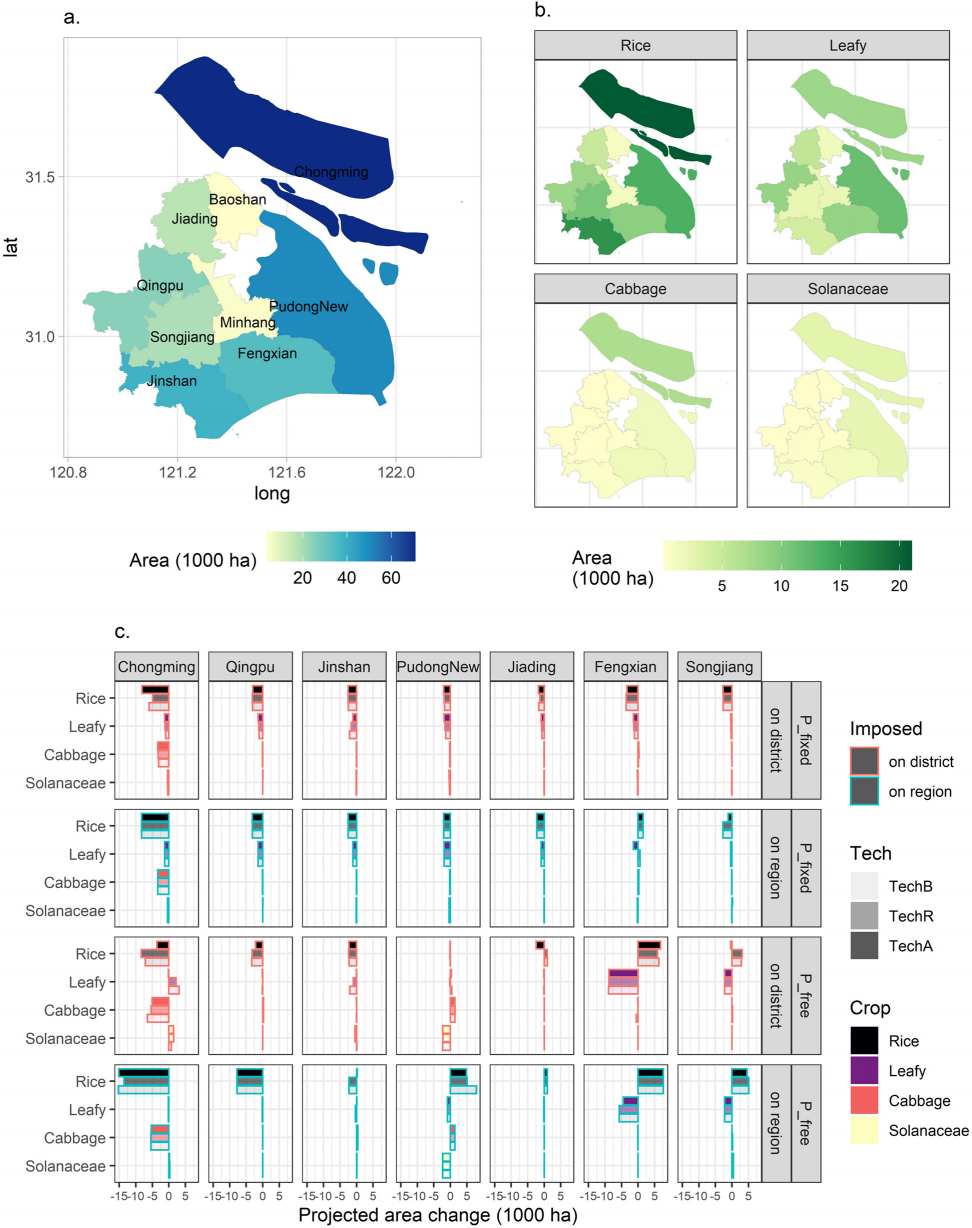
**District-level crop areas and projected changes under the 20% reduction scenarios by the pesticide application method across peri-urban Shanghai.** (a) Base district-level crop area totals in Shanghai, 2015. Low-mid-high color gradient is used. Chongming shows the largest acreage of 60+ thousand ha, while inner suburban districts (Baoshan and Minhang) exhibit the lowest acreages. (b) Base (2015) district-level distribution of areas for major representative crops (rice, leafy, cabbage, and Solanaceae). Rice production primarily takes place in Chongming, Jinshan, and PudongNew (eastern). Leafy greens mainly in PudongNew. Solanaceae mainly in Chongming and PudongNew. (c) Projected area changes for select crops by district under various scenarios. To save space and for clarity, only R3 (20% reduction in pesticide use) scenarios are used. Districts experiencing virtually zero changes in crop areas (Baoshan and Minhang) are not displayed either. From left to right, districts are ordered according to the magnitudes of their overall net area changes. Chongming sees decreases in crop areas. In particular, compared with district-level imposition, the regionwide imposition scenarios that allow flexibility in cross-district flows result in larger reductions in rice areas for Chongming. Meanwhile, larger flow-ins of rice areas are shown for PudongNew and Fengxian. The “P_free” closed agricultural economy assumptions would reinforce this pattern. Full versions of panels (b) and (c) are provided in Supplemental File S3 (Figs. S3 and S4).

Despite the changes discussed above, one may note that districts with large decreases in the total pesticide use (Fig. 1c), such as Chongming, exhibited relatively stable use intensity levels across the different scenarios (Fig. 2c). This implies that pesticide use reductions may have been achieved via downsizing crop acreages. Fig. 3c presents the decomposition of the district-level changes in the crop mix, focusing on the 20% (R3) reduction scenarios.

District-wise, the largest decline in crop area and hence crop production was observed in Chongming (northern islands), followed by Qingpu (westernmost) and Jinshan (southernmost), which are major agricultural suburban districts. However, crop-planted areas would shift towards Fengxian (south coastal), PudongNew (eastern), and Songjiang (southwestern). Additionally, for Minhang (central) and Baoshan (northern) not presented in Fig. 3c, only slight changes in the crop mix occurred in these two most industrialized peri-urban districts in the Shanghai area.

Crop-wise, rice exhibited the greatest crop-specific movements, shifting away from the islands in North Shanghai (Chongming: -15.30 to -3.61 kha) and West Shanghai (Qingpu: -7.79 to -2.16 kha, under various R1-R5) toward the east, especially under the “closed agricultural economy” and “imposed on region” combination scenarios that implicitly encouraged and allowed more room for regional reallocation. However, leafy vegetables exhibited decreases in most major production districts. The exception was that with “imposed on district”, leafy vegetables incurred expansions in Chongming, implying that the district-level imposition displayed greater impacts on the production of pesticide-intensive crops. Note that both rice and leafy vegetables occupy the most agricultural land in the Shanghai area (Fig. 3b). Besides, the cabbage area decreased in Chongming, but rose noticeably in PudongNew. The area of Solanaceae (such as tomato (*Solanum lycopersicum*)) experienced the opposite, seeing increases in Chongming and declines in PudongNew, indicating the regional heterogeneity in the competitiveness of crop enterprises.

Technology-wise, the “rice only” (TechR) adoption of chemicals application machinery would modify the changes varying between crops mentioned above, largely ameliorating the shifts in acreages. The most noteworthy modifications occurred for rice in Chongming and PudongNew under the “P_free + regionwide imposition” scenarios. The “all-crops” (TechA) adoption approach however did not diminish the regional and crop heterogeneity effects of reductions much in general.

Overall, aggregating the crop-specific acreage shifts shows that, the peri-urban-wide reduction plan would primarily target Chongming, which exhibited the highest total pesticide usage among the districts, and Qingpu, which exhibited the greatest pesticide use intensity.

### 3.3 Impacts on food security in terms of physical quantity and price

**Fig. 4:**
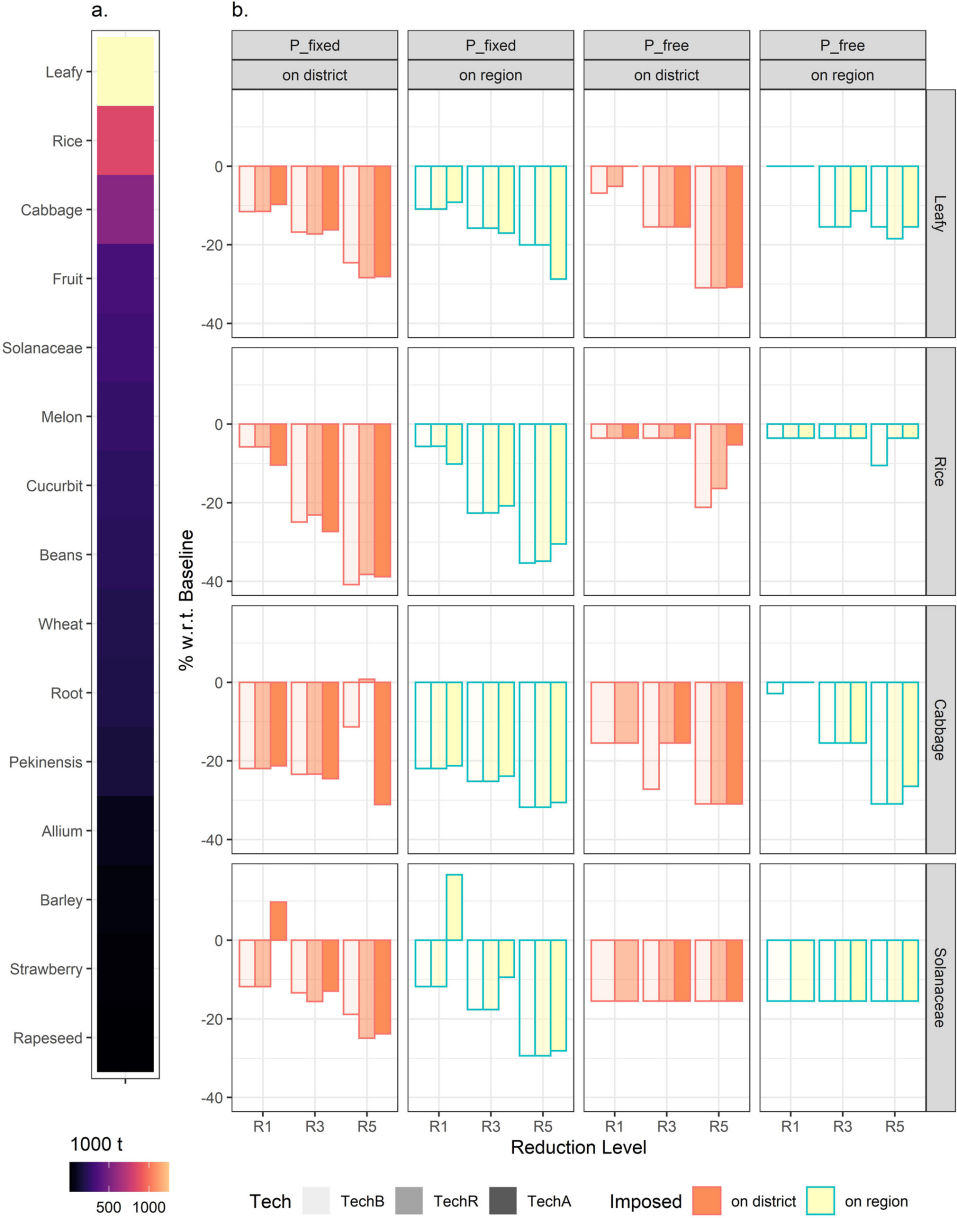
**Levels of and projected changes in crop production under various scenarios.** (a) Base production levels by crop, 2015. Leafy vegetables and rice are the top two crops produced in Shanghai. (b) Projected percentage changes in production levels with respect to baseline scenarios by crop under different scenarios. To save space and for clarity, only major representative crops (leafy, rice, cabbage, and Solanaceae) are shown. Also, only R1, R3, and R5 (indicating 10%, 20%, and 30% reduction in pesticide use levels, respectively), are used. Compared with “P_free” closed agricultural economy scenarios, the “P_fixed” price-taker scenarios exhibit much larger losses in crop production levels, especially for leafy vegetables and rice. The district-level imposition brings about larger reductions than the regionwide imposition in general. The exception is that with TechA (chemicals application machinery is applicable for all crops), the production level of Solanaceae may see rises under mild reduction level (R1) scenarios. A full version of panel (b) is provided in Supplemental File S3 (Fig. S5).

The reduction scenarios had food security effects. Herein we examined the two aspects regarding food security: physical availability and affordability. As shown in Fig. 4b, the production of leafy vegetables (mainly consisting of Pak Choi (*Brassica chinensis*)), as the primary local produce (Fig. 4a), substantially declined under the reduction goals (-17.26% to -11.39%, for various R3 scenarios), especially for the district-level reduction imposition scenarios. Similarly, rice production decreased (-27.31% to -3.54% under various R3) when reduction mandates were implemented, with a large reduction under the 30% goal (R5).

If pesticide-saving technologies were adopted, then both leafy vegetables and rice production were less affected. Cabbage (*Brassica oleracea*) production was more severely affected (-27.19% to -15.46% under R3). Solanaceae saw varying changes (-17.65% to -9.39% under R3) including increases (+16.68% under R1+TechA) in their production levels. This reflects economic competitiveness associated with these enterprises, and such an advantage outweighs the disadvantages of pesticide-intensive farming when the regionwide or district-level reduction mandates were not tightened (e.g. a 10% reduction under R1).

**Fig. 5:**
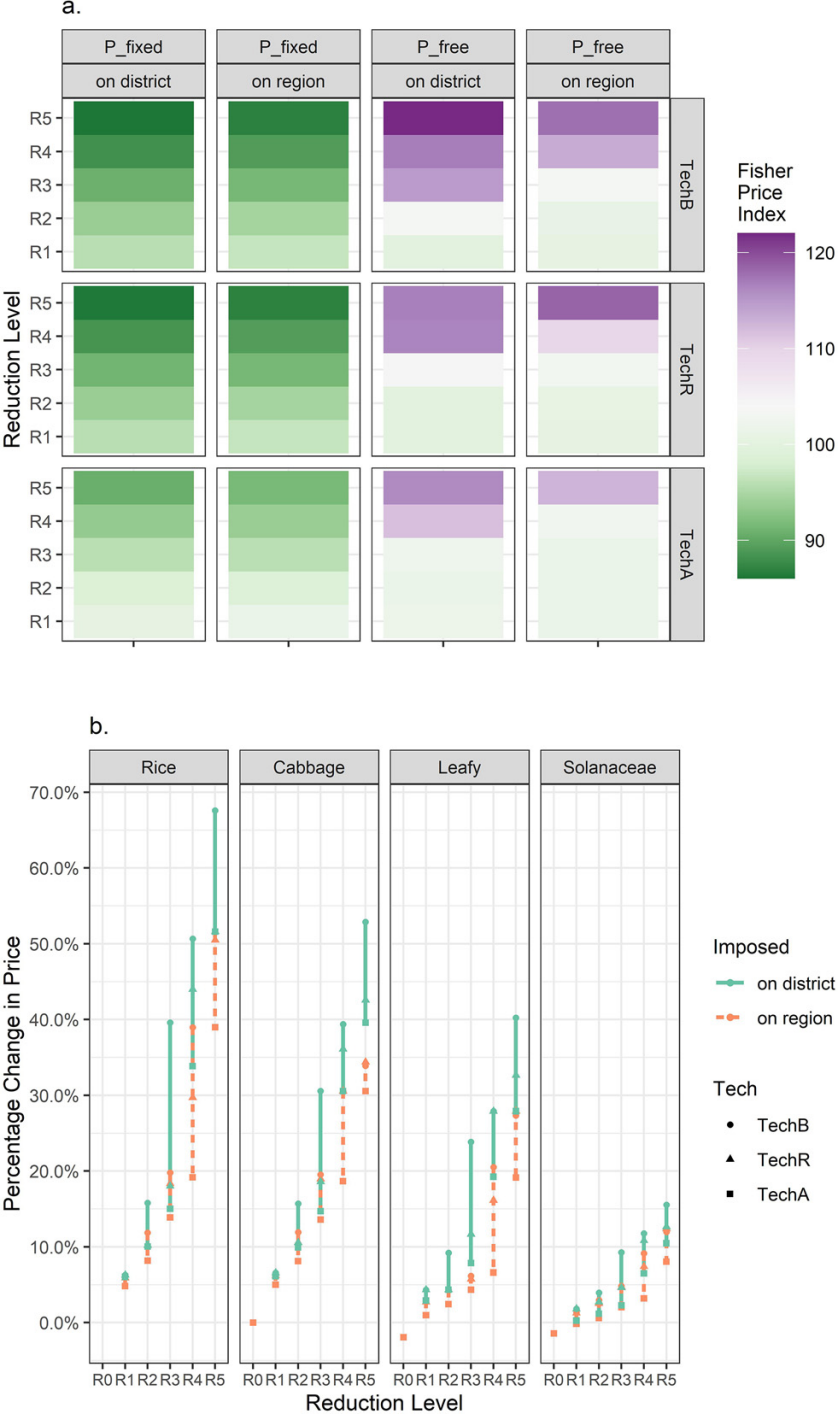
**(a) Projected agricultural Fisher price index and (b) various agricultural product price percentage changes under different scenarios.** (a) Projected Fisher price index values under various scenarios. Diverging color gradient is used to indicate more or less than the baseline 100+/- levels. Under “P_fixed” price-taker assumptions, the Fisher price index sees decreases, implying crop production decreases in general. With TechA that allows all crops to have the possibility of adopting chemicals application machinery, milder reductions are expected. This implies the proportion of high-priced crops gets slightly larger under TechA scenarios. The “P_free” closed agricultural economy assumptions result in much larger Fisher index values, reflecting the underlying price increases. The district-level imposition that restricts cross-district flows of pesticide use reduction works can further raise the Fisher price index. (b) Projected percentage changes in prices by crop under various “P_free” scenarios. To save space and for clarity, only major representative crops (leafy, rice, cabbage, and Solanaceae) are used. Rice sees the most pronounced price changes among the selected crops. In general, the district-level imposition notably raises the price levels, and TechR and TechA that allow the possibility of sprayer machinery adoption would modulate the changes. A full version of **b** in absolute price is provided in Supplemental File S3 (Fig. S6).

It presents the alterations in the summary Fisher price index values (Fig. 5a) and individual crop prices (Fig. 5b) as induced by various P_free scenarios that generate endogenous prices. Generally, crop prices followed increasing trends as the reduction requirements became more stringent (100.68-117.79, under TechB R1-R5), and the district-level imposition (100.01-121.99, under TechB R1-R5) significantly raised the price levels than the regionwide imposition. These increases in the price and index value corresponded to decreases in the production levels, as discussed above.

The adoption of pesticides application machinery reduced the price increases, with the “all crops” (TechA) scenario exhibiting greater diminishing effects than the “rice only” (TechR) case. Moreover, the ameliorating effects of “adoption” on prices were more apparent in terms of percentage changes when the reduction goals exceeded 20%, particularly for field crops such as rice (TechB: 19.80% to 51.60%; TechR: 18.36% to 50.50%; TechA: 13.89% to 38.98%, under R3-R5 with the regionwide imposition).

The notable variations in the changes in crop production levels and prices, implied that reductions in crop production or cropping land use had been drivers to help materialize pesticide usage reduction. The production of leafy vegetables was the most affected due to their comparatively advantageous production costs, as well as the market preferences that reflect how consumers would respond to the price changes in vegetable crops.

## 4 Discussion

Although the main purpose of applying pesticides is controlling damage to agricultural production, using or overusing pesticides has significant environmental implications, causing soil and aquifer contamination [[4], [6]] and affecting aquatic ecosystems [49]. Much of the research conducted in China focused on legacy organochlorine insecticides, such as dichloro-diphenyl-trichloroethane (DDT) and hexachlorocyclohexane (HCH), while currently used pesticides have received limited attention [50]. In metropolitan areas, such as Shanghai with 24.87+ million residents situated in the densely populated East China, surface runoff from peri-urban agricultural production is the main source of non-point source water pollution [51]. Moreover, as Shanghai sits on the easternmost of the Yangtze River Delta and that the Yangtze River outflow is recognized as a major secondary source of organochlorine pesticides (OCPs) for the East China Sea [52], it is beneficial to put Shanghai's agricultural pesticide use under control. Meanwhile, the Dianshan Lake, serving as a drinking water source located in the east of Shanghai, faces water quality deterioration with pesticides being among the main types of pollutants [[53], [54]]. Besides, agricultural pesticides have contributed to the degradation of soil quality in the Shanghai Region via heavy metal deposition [55].

According to the projected pesticide usage totals, decreases accompanied the decreases in crop acreage for the Chongming, Qingpu, and Jinshan Districts (Fig. 1b and Fig. 3c). However, the usage intensity (kg/ha) did not necessarily change in the same direction. This lends support to why research interest in farmer behaviors of pesticide use (more related to a per unit of land basis) has persisted till today, and that extensive margins are worthy of attention when designing and evaluating pesticide policies [28]. From a prevention perspective, the decreases imply that the non-point source pollution (NPS) pressure on soil and waterbodies in the Dianshan Lake, west of Shanghai (Qingpu), and the Yangtze River Estuary in the North (Chongming), would be reduced (see Fig. 1a). Compared with the case study of a surface water catchment located in the UK [29], in which mitigation coordination appeared to be demanding for farmers, this study suggested that on a broader scope, a reduction in total pesticide use could be facilitated at a broader level via the mediation of agricultural commodity markets.

**Fig. 6:**
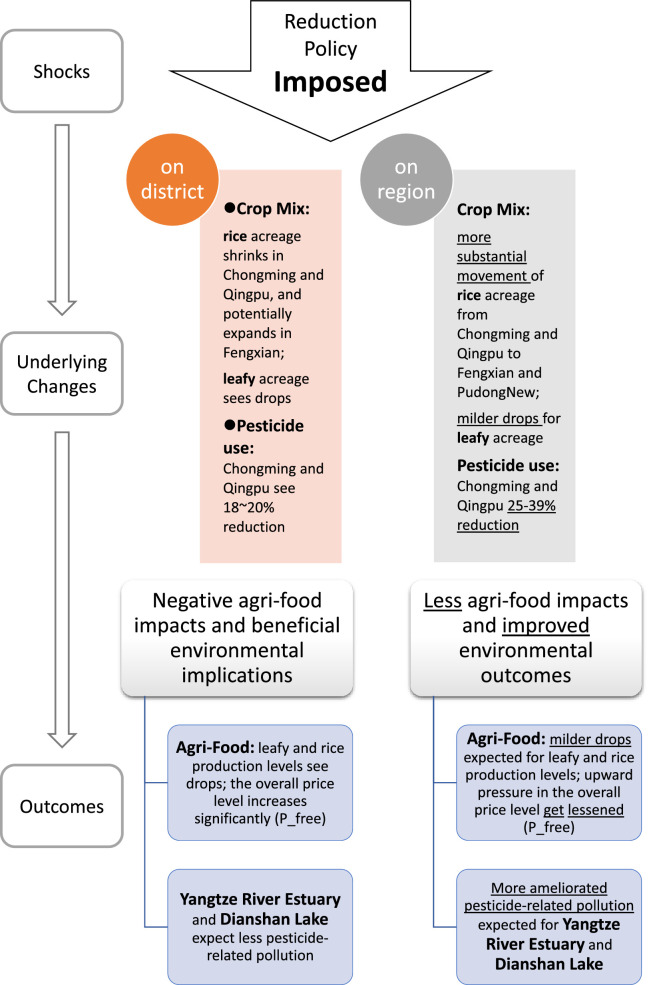
**Comparison of the district- and region-level imposition of the Shanghai pesticide use reduction policy.** Qualitative differences in the underlying changes (crop mix and pesticide use) and outcomes (agri-food impacts and the pesticide pollution implications) under R3 (20% reduction) are exhibited here. The effects of the region-level imposition with respect to the district-level are in particular underscored.

Moreover, the comparison of imposing pesticide use reductions at the district-level and the region-level (Fig. 6) reveals that the more disaggregated mandates would cause more severe consequences for local food security, with significantly higher prices and larger declines in production of important daily consumed commodities such as rice, leafy vegetables, and cabbage. Enabling regionwide adjustments however induces a greater proportion of the adaptation pressure to occur in major agricultural districts, especially Chongming and Qingpu (Fig. 3c). These two districts would commit greater than prescribed rates of pesticide use reduction (Fig. 1c) and also likely bring about larger NPS prevention benefits. With the possibility of regionwide adjustments, rice shall be the most affected crop experiencing notable acreage movements between districts, whereas with the district-level requirements, both rice and vegetables (especially leafy ones and cabbage) incurred noteworthy alterations within the district. Simply put, one may find that a reduction plan that enables regional adjustments may ameliorate the negative impacts on local crop production and upward pressure on price, meanwhile generating more possible NPS benefits for the Yangtze River Estuary and the Dianshan Lake that bear important ecological and environmental significance. This suggests that when the usage mandate is implemented at appropriate scale, both agri-food benefits and improved environmental outcomes could be simultaneously obtained, rather than presenting a trade-off [56] as is often the case discussed in existing literature.

Comparing the projected reductions in leafy vegetables and rice production under R5 (30%) with the official statistics (-37.24% for pesticide use) provided in Section 1, leafy (-28.75% to -20.05% under P_fixed+R5 vs. -26.37% for vegetable production) exhibited much smaller deviations than rice (-40.81% to -30.51% under P_fixed+R5 vs. -14.29% for staple crops). With the assumptions under P_free, rice production then saw decreases with remarkably smaller magnitudes (-21.19% to -3.54%). Leafy would however incur larger variations (-30.93% to -15.46%). These differences imply that consumers in Shanghai do have preferences over local produce to certain extent. And the use of hypothetical scenarios assuming a closed regional agricultural economy by this study gains more legitimacy. Additionally, such decreases, whether recorded or projected, remained substantial, raising concerns over local food security. Therefore, technologies that can reduce pesticide application, particularly for rice and leafy vegetables, are required if food security were to remain as intact as possible when operating pesticide reduction policies.

## 5 Conclusion

By applying a newly developed regional agricultural sector model, this study explored the effects of pesticide use reduction policy on Shanghai's peri-urban agricultural sector from multiple aspects. Herein we find that achieving the 20% reduction goal exhibited the largest effects on the districts with high pesticide use totals and/or intensities (i.e., Chongming and Qingpu), causing notable crop mix changes and potentially reducing pesticide non-point source pollution for the Yangtze River Estuary and the Dianshan Lake. Among the different crop enterprises, the local production of rice and leafy vegetables would be most negatively affected, seeing projected declines of -22.62% and -15.74%, respectively. Mechanizing chemicals application that allows more precise pesticide use can modulate the impacts. Moreover, imposing the reduction requirements at district-level may aggravate local food security concerns, and reduce environmental benefits. Furthermore, a closed Shanghai agricultural economy that incorporates strong preference for local produce would enlarge the disparity between districts in the above-mentioned outcomes.

This study is subject to at least a few limitations. First, the sector-optimization model has focused on local produce, considering two extreme cases. One describes the more realistic situation in which Shanghai acted as a price taker, while the other adopts a hypothetical case assuming a closed Shanghai's agricultural economy. More detailed assumptions regarding consumers’ stickiness to local produce shall help the model to better represent the demand side. After all, it remains a global challenge to localize food systems that provide sufficient amounts of nutrients for dense populations [57]. Second, there are three main channels for reducing pesticide usage, i.e., the reallocation of inputs, increasing application precision, and changing the crop mix. This study focused on the “altering crop mix” and “increasing precision” channels only. Other “super extensive” margins [28] could be considered, such as introducing frogs (*Rana*) [58] and ducks (*Anas*) [59] as forms of insect control, as well as “rice-shrimp” coculture [60], which has minimal pesticide usage. These alternatives would all lead to lower pesticide residue levels. Third, more informative NPS implications such as in Kudsk et al. [10] could have been derived, should a pesticide transport model were incorporated or linked to the SH-ASM. Future research may include additional pesticide use reduction alternatives mentioned above, and derive the nuances needed to answer how practices may affect the transport and fate of pesticides [61]. Fourth, this study did not consider significant disruptive cases such as large-scale armyworm invasion [62], which may render the increases in pesticide use inevitable. Future research may need to incorporate the component of uncertainty analysis that takes both business-as-usual variation on the supply side [57] and extreme events into consideration. Additionally, the GAMS-written model and the data used in this study may potentially be employed and adapted for the investigation of peri-urban agri-environmental issues worldwide, even though new development efforts would be required for other metropolitan areas [63].

## Data availability

The major data used as model inputs to the Shanghai Agricultural Sector Model are available in *figshare* with the identifier doi: 10.6084/m9.figshare.16421727.v2 [34]. The data on the district- and crop-specific inputs usage would be available upon reasonable request to Y.W.Z. The data that support the findings of this study are also available in *figshare* with the identifier doi: 10.6084/m9.figshare.16423599.v2 [64]. Fig. 1, Fig. 2, Fig. 3, Fig. 4, Fig. 5 (main text) and S1-S6 (S3) are associated with these data.

## Code availability

The core GAMS codes of the Shanghai Agricultural Sector Model are available in *figshare* with the identifier doi: 10.6084/m9.figshare.16421727.v2 [34]. The full version of the GAMS codes would be available upon reasonable request to Y.W.Z. Also, the R codes for generating the figures (main text) are available in *figshare* with the identifier doi: 10.6084/m9.figshare.16423365.v2 [65].

## Ethical compliance

The survey carried out in this study does not require institutional ethical approval according to the guidelines and policies set out by the Science and Technology Ethics Committee of Shanghai Jiao Tong University. Informed consent was obtained from all the participants.

## Declaration of interests

The authors declare that they have no conflicts of interest in this work.

## Appendix Supplementary materials

Image, application 1

Image, application 2

Image, application 3
