# Supplementary material for: Operating pesticide use reduction within the boundary of food security in peri-urban settings
Source: Fundam Res. 2022 Apr 18;2(4):635–47. doi: 10.1016/j.fmre.2022.04.003 (PMC11197716; doi:10.1016/j.fmre.2022.04.003)
Supplement: Supplementary file 1 [file mmc1.docx]

## Supplemental File S1

Part A. GAMS Code of “Model Structure”

Part B. Model Calibration

## Part A. GAMS Code of “Model Structure” in Shanghai Agricultural Sector Model (SH-ASM)

*-------------------------------------------------------------------------------

* Core Model

*-------------------------------------------------------------------------------

POSITIVE VARIABLES

AGDEMAND(CROPS) demand curve for crop

AGDEMANDS(CROPS, INCREMENT) demand curve in step for crop -- lamdas

CROPBUDALT(CROPS, REGIONS, TECHS) land allocation for crop|region|tech

AGMIX(YEARS) convex historical mix for crop category -- alphas

AGMIXSE(YEARS) convex staple-econ adjusted historical mix for crop -- gammas

$ifthen setglobal use_artificial

ARTCROPBUDALT(CROPS, REGIONS, TECHS) artificial variables for cropbudalt

$endif

;

VARIABLES

WELFARE total market welfare for Shanghai crop commodities

;

EQUATIONS

WELFARE_OBJ objective function - welfare maximization

COMMODITYBAL(CROPS) supply and demand balance - for all crops

LANDBAL(REGIONS) land balance - for all ag-related regions

INPUTBAL(ITEMS) input usage overall balance - for all items physical

DEMANDIDENTITY(CROPS) sum of agdemands X qinc = agdemand - for all crops

DEMANDLOWER(CROPS) lower bound for agdemand - for all crops

DEMANDCONVEXITY(CROPS) convexity constraint for demand - for all crops

LANDMIXLO(STAPLEECON) historical crop mix constraint - for cateogries - lower

LANDMIXUP(STAPLEECON) historical crop mix constraint - for categories - upper

LANDMIXCONVEXITY convexity constraint for cropland mix

LANDMIXSELO(REGIONS, CROPS) staple-econ adjusted crop mix constraint - lower

LANDMIXSEUP(REGIONS, CROPS) staple-econ adjusted crop mix constraint - upper

LANDMIXSECONVEXITY convexity constraint for staple-econ adjusted crop mix

;

WELFARE_OBJ..

WELFARE =E=

*--- demand curve --- pay attention to units --- demand_points("quantity") in 10X4 Ton whereas cropbud("quantity") in Kg ---

*--- demand curve --- pay attention to units --- demand_points("price") in RMB per Kg and cropbud("price") in RMB per Ha ---

SUM((CROPS, INCREMENT),

DEMAND_POINTS(CROPS, "P_hat")*PHAT_ADJUST(CROPS)

/(1+1/DEMAND_POINTS(CROPS, "Elast"))*

(QINC(INCREMENT)**(1+1/(DEMAND_POINTS(CROPS, "Elast"))))*

AGDEMANDS(CROPS, INCREMENT))

*--- supply curve | production cost ---

-SUM((CROPS, REGION, TECHS), SUM(ItemsCost,

$ifthen setglobal use_artificial

(ARTCROPBUDALT(CROPS, REGION, TECHS)+CROPBUDALT(CROPS, REGION, TECHS))*

$else

CROPBUDALT(CROPS, REGION, TECHS)*

$endif

CropBudgetAlt(Crops, Region, Techs, ItemsCost)*InputUnitCost(ItemsCost, "2015")))

$ifthen setglobal use_artificial

-SUM((CROPS, REGION, TECHS), (ARTCROPBUDALT(CROPS, REGION, TECHS)+CROPBUDALT(CROPS, REGION, TECHS))*

CropBudgetAlt(Crops, Region, Techs, "Implicit"))

$else

-SUM((CROPS, REGION, TECHS), CROPBUDALT(CROPS, REGION, TECHS)*CropBudgetAlt(Crops, Region, Techs, "Implicit"))

$endif

$ifthen setglobal use_artificial

-SUM((CROPS, REGION, TECHS), (10**9)*ARTCROPBUDALT(CROPS, REGION, TECHS))

$endif

;

COMMODITYBAL(CROPS)..

*--- pay attention to units -- agdemand perhaps in 10X4 ton whereas cropbudget in Kg per Ha ---

AGDEMAND(CROPS) =L= SUM((REGION, TECHS),

$ifthen setglobal use_artificial

(ARTCROPBUDALT(CROPS, REGION, TECHS)+CROPBUDALT(CROPS, REGION, TECHS))*

$else

CROPBUDALT(CROPS, REGION, TECHS)*

$endif

CropBudgetAlt(Crops, Region, Techs, Crops));

LANDBAL(REGION)..

$ifthen setglobal use_artificial

SUM((CROPS, TECHS), ARTCROPBUDALT(CROPS, REGION, TECHS)+CROPBUDALT(CROPS, REGION, TECHS))

$else

SUM((CROPS, TECHS), CROPBUDALT(CROPS, REGION, TECHS)

$endif

=L= AreaRegion(Region);

INPUTBAL(ITEMS)$SUM(YEARS, INPUT_SH_BASIC(ITEMS, YEARS))..

SUM((CROPS, REGION, TECHS),

$ifthen setglobal use_artificial

(ARTCROPBUDALT(CROPS, REGION, TECHS)+CROPBUDALT(CROPS, REGION, TECHS))*CropBudgetAlt(Crops, Region, Techs, Items))

$else

CROPBUDALT(CROPS, REGION, TECHS)*CropBudgetAlt(Crops, Region, Techs, Items))

$endif

=L= INPUT_SH_BASIC(ITEMS, "2015")*(10**4)*(10**3);

DEMANDIDENTITY(CROPS)..

*--- pay attention to units -- agdemand perhaps in 10X4 ton whereas demand_points in 10X4 ton

AGDEMAND(CROPS) =E= SUM(INCREMENT,

QINC(INCREMENT)*DEMAND_POINTS(CROPS, "Q_hat")*AGDEMANDS(CROPS, INCREMENT));

DEMANDLOWER(CROPS)..

SUM(INCREMENT,

QINC(INCREMENT)*DEMAND_POINTS(CROPS, "Q_hat")*AGDEMANDS(CROPS, INCREMENT)) =G= 1/10*DEMAND_POINTS(CROPS, "Q_hat");

DEMANDCONVEXITY(CROPS)..

SUM(INCREMENT, AGDEMANDS(CROPS, INCREMENT)) =E= 1.00;

LANDMIXLO(STAPLEECON)..

SUM(STAPLEECON_MAP(STAPLEECON, CROPS),

$ifthen setglobal use_artificial

SUM((REGION, TECHS), (ARTCROPBUDALT(CROPS, REGION, TECHS)+CROPBUDALT(CROPS, REGION, TECHS))))

$else

SUM((REGION, TECHS), CROPBUDALT(CROPS, REGION, TECHS)))

$endif

=G= AGMIX_LO*SUM(YEARS$AreaSH_Mix(Years, "Total"), AGMIX(YEARS)*AreaSH_Mix(Years, StapleEcon));

LANDMIXUP(STAPLEECON)..

SUM(STAPLEECON_MAP(STAPLEECON, CROPS),

$ifthen setglobal use_artificial

SUM((REGION, TECHS), (ARTCROPBUDALT(CROPS, REGION, TECHS)+CROPBUDALT(CROPS, REGION, TECHS))))

$else

SUM((REGION, TECHS), CROPBUDALT(CROPS, REGION, TECHS)))

$endif

=L= AGMIX_UP*SUM(YEARS$AreaSH_Mix(Years, "Total"), AGMIX(YEARS)*AreaSH_Mix(Years, StapleEcon));

LANDMIXCONVEXITY..

SUM(YEARS$AreaSH_Mix(Years, "Total"), AGMIX(YEARS)) =E= 1.00;

LANDMIXSELO(REGION, CROPS)..

$ifthen setglobal use_artificial

SUM(TECHS, (ARTCROPBUDALT(CROPS, REGION, TECHS)+CROPBUDALT(CROPS, REGION, TECHS)))

$else

SUM(TECHS, CROPBUDALT(CROPS, REGION, TECHS))

$endif

=G= AGMIXSE_LO*SUM(YEARS$AreaRegion_MixSE(Region, Crops, Years), AGMIXSE(YEARS)*AreaRegion_MixSE(Region, Crops, Years));

LANDMIXSEUP(REGION, CROPS)..

$ifthen setglobal use_artificial

SUM(TECHS, (ARTCROPBUDALT(CROPS, REGION, TECHS)+CROPBUDALT(CROPS, REGION, TECHS)))

$else

SUM(TECHS, CROPBUDALT(CROPS, REGION, TECHS))

$endif

=L= AGMIXSE_UP*SUM(YEARS$AreaRegion_MixSE(Region, Crops, Years), AGMIXSE(YEARS)*AreaRegion_MixSE(Region, Crops, Years));

LANDMIXSECONVEXITY..

SUM(YEARS$SUM((Region, Crops), 1$AreaRegion_MixSE(Region, Crops, Years)),

AGMIXSE(YEARS)) =E= 1.00;

$ifthen setglobal use_cropbud_baseonly

CROPBUDALT.FX(CROPS, REGION, TECHS)$(NOT SAMEAS(TECHS, "base")) = 0.00;

$endif

*-------------------------------------------------------------------------------

* Model Statement

*-------------------------------------------------------------------------------

MODEL SH_AG_MOD /ALL/;

SOLVE SH_AG_MOD USING LP MAXIMIZING WELFARE;

*-------------------------------------------------------------------------------

*-------------------------------------------------------------------------------

## Part B. Model Calibration

### P_free version

To let the model generate baselines that are closer to the observed prices $\hat{P}$ and quantities $\hat{Q}$, we adopted the following “iterative” calibration approach.

First, we calculate the ratios of the simulated prices (the shadow prices of commodity supply-demand balance equations) over the observed prices, and then use these ratios as adjusting factors, inserting them into the demand function in the objective equation. After solving the model, we then compare the newly simulated quantities with the observed quantities.

The adjusting factors are calculated using the following equations. The purpose is that we try to obtain factors that can help generate equilibrium quantities and prices that deviate away from the observed equilibriums as little as possible.

$$\hat{P}_{adjust,j,P}={\hat{P_{j}}}/{P_{tmp,j}}$$

$$\hat{P}_{adjust,j,Q}=\left[ {\hat{Q}_{j}-Q_{tmp.j})}/{Q_{tmp,j}} \right]/{\varepsilon_{j}}+1$$

where $\varepsilon_{j}$ refers to the constant price elasticity of demand for commodity $j$.

Thereafter, we obtain the newly simulated prices, and compare them with the observed prices. And then we shift attention to quantities.

Adopting the iterative $P\to Q\to P\to Q\to\ldots$ calibration process, we aim to replicate the observed baselines as much as possible.

The terminal conditions are as follows.

$$\max_{j} \left| {Q_{tmp,j}}/{\hat{Q}_{j}}-1 \right|\leq0.20$$

$$\max_{j} \left| {P_{tmp,j}}/{\hat{P}_{j}}-1 \right|\leq0.20$$

### P_fixed version

For the P_fixed case, the model would then set the constant price elasticity of demand to negative infinity.

$$\varepsilon_{j}=-\infty, \forall j$$

We would then obtain the shadow prices of the commodity balance equations. Compare the estimated prices with the recorded prices to obtain the adjusting factors, as below.

$$\hat{P}_{adjust,j,P}={\hat{P_{j}}}/{P_{tmp,j}}$$

Unlike the P_free case, where would be Q-based adjusting factors, the P_fixed case relies on P-based adjusting factors only. Note that the constant price elasticity of demand is set to negative infinity for all crops.

Besides, barley is a special case when calibrating the “P_fixed” case of the model. Due to its lack of economic competitiveness, the simulated production level had been slightly below 80% of the recorded level. Therefore, an arbitrary lower bound condition as below was added to the model structure.

$$Q_{tmp,Barley}\geq0.80*\hat{Q}_{Barley}$$

The iterative $P\to P\ldots$ adjusting process would keep going on until the following conditions are met.

$$\max_{j} \left| {Q_{tmp,j}}/{\hat{Q}_{j}}-1 \right|\leq0.20$$

### Caveats

Cabbage: compared with Pekinensis, cabbage yields are relatively lower across the districts of Shanghai. With similar prices, it would be more likely to see land allocated to Pekinensis than cabbage under an optimization framework.

Allium: with widely ranging yields across the districts and a relatively lower price level among different kinds of vegetables, allium production tends to get allocated to its lower yielding districts such as Chongming, under an optimization framework.

The above implies that the areas of cabbage and allium would see upwardly biased estimates. This is a systematic bias associated with this model.
