# Supplementary material for: Operating pesticide use reduction within the boundary of food security in peri-urban settings
Source: Fundam Res. 2022 Apr 18;2(4):635–47. doi: 10.1016/j.fmre.2022.04.003 (PMC11197716; doi:10.1016/j.fmre.2022.04.003)
Supplement: Supplementary file 2 [file mmc2.docx]

## Supplemental File S2

Part A. Questionnaire

Part B. History

## Part A. Questionnaire of Shanghai Farming Household Survey (2020)

Dear Sir/Madam,

Greetings from a research project team from Shanghai Jiao Tong University. Our team is working on *Scenarios and Policy Analysis of Smart Agricultural Machinery Adoption among Small and Mid-Sized Farming Households in Shanghai*. To help improve total efficiency and labor productivity for modern urban agriculture in Shanghai, and to provide policy support for the construction of agricultural big data, we are carrying out research regarding the behaviors of small and mid-sized farming households regarding the allocation of economic resources and the associated environmental conditions in the suburban areas of Shanghai. The questionnaire is to be filled anonymously, and all the information collected will be used for statistical and scientific analysis only. Your data will be kept strictly confidential, so please feel free to fill in the questionnaire according to your actual situation. Thank you for your support and help!

1. How many mu (1 ha=16 mu) of land does your family farm own? Among which, how many mu are allocated for rice production, and how many seasons are planted a year? If other crops are also grown, the main crop is (single choice): wheat, barley, melon, rapeseed, strawberry, fruits, leafy vegetables, Chinese cabbage (pekinensis), cabbage, root vegetables (radish/potato, etc.), cucurbit (cucumber), beans, Solanaceae, allium? And how many seasons are planted in a year?
2. How many mu of land does your family farm contract? How many mu of land are transferred in? How much is the average transfer cost per mu？
3. What is your family farm's rice yield in 2019 (kg/mu, single choice: <450, ≥450 & <500, ≥500 & <550, ≥550 & <600, ≥600)? What is the total output of other crops (if it is grain or oil crops, kg/mu, single choice: <200, ≥200 & <300, ≥300 & <400, ≥400 & <500, ≥500; if it is vegetables or fruits, kg/mu, single choice: <1000, ≥1000 & <2000, ≥2000 & <3000, ≥3000 & <4000, ≥4000)?
4. Does your family farm possess own agricultural machinery? If so, what are the primary uses of own machinery (multiple choices: land preparation, sowing, chemicals application, harvesting)? How much is the horsepower (single choice: <25, ≥25 & <40, ≥40 & <60, ≥60 & <80, ≥80)? How much did the agricultural machinery cost (ten thousand yuan, single choice: <1, ≥1 & <2, ≥2 & <3, ≥3 & <4, ≥4)? What about your proficiency in operating the machinery (single choice: very proficient, relatively proficient, average, not proficient, not able to operate)?
5. Does your family farm rent agricultural machinery? If so, what are the primary uses of rented agricultural machinery (multiple choices: land preparation, sowing, chemicals application, harvesting)? How much is the horsepower (single choice: <25, ≥25 & <40, ≥40 & <60, ≥60 & <80, ≥80)? How much is the rent cost (yuan/mu, single choice: <100, ≥100 & <150, ≥150 & <200, ≥200 & <250, ≥250)?
6. As shown in the figure below, have you ever used an "smart rice seed planter"? If not, have you ever seen or heard about it before?


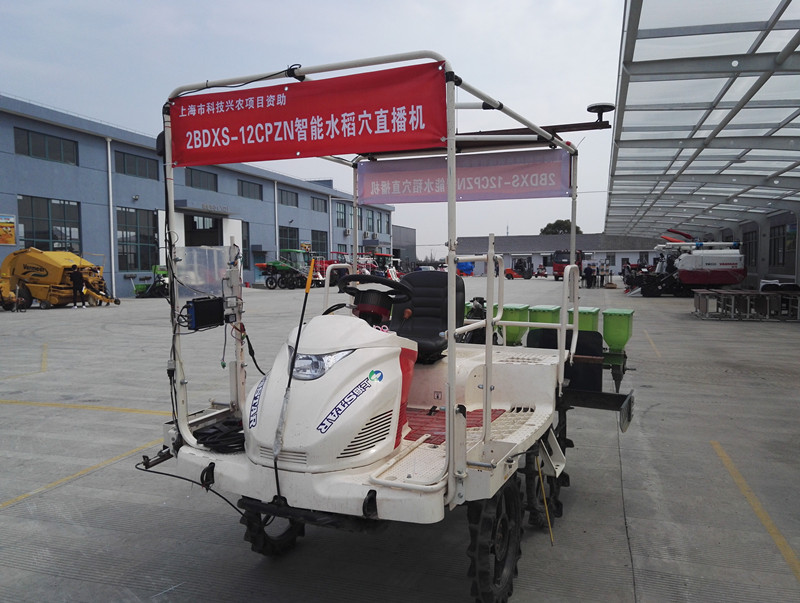


Figure S2-1. Smart Rice Seed Planter.

Figure Source: <http://me.sjtu.edu.cn/news/11584.html> http://me.sjtu.edu.cn/news/11584.html

1. How many people from your family do you need for growing rice in 2019? How many months does everyone work on average (months, single choice: <2, ≥2 & <4, ≥4 & <6, ≥6 & <8, ≥8)? If you hire workers, how many workers do you need to hire? On average, how many months does each worker work (months, single choice: <2, ≥2 & <4, ≥4 & <6, ≥6 & <8, ≥8)? How much do you pay for the workers (yuan/person·month, single choice: <1000, ≥1000 & <2000, ≥2000 & <3000, ≥3000 & <4000, ≥4000)?
2. Besides land, machinery and labor, how much do other inputs (seeds, fertilizers, pesticides) cost for growing rice (yuan/mu, single choice: <250, ≥250 & <500, ≥500 & <750, ≥750 & ≤1000, >1000)? Among these inputs, how much chemical fertilizers do you use (kg/mu, single choice: <20, ≥20 & <40, ≥40 & <60, ≥60 & ≤80, >80), and how much do chemical fertilizers cost (yuan/mu, single choice: <50, ≥50 & <75, ≥75 & <100, ≥100 & ≤125, >125)? How much pesticides do you use (kg/mu, single choice: <0.5, ≥0.5 & <0.75, ≥0.75 & <1.0, ≥1.0 & ≤1.25, >1.25), how much do pesticides cost (yuan/mu, with single choice: <10, ≥10 & <15, ≥15 & <20, ≥20 & ≤25, >25)?
3. Do you grow other crops besides rice? Did you purchase agricultural machinery yourself for growing other crops, and if so, how much does the machinery cost (ten thousand yuan, single choice: <1, ≥1 & <2, ≥2 & <3, ≥3 & ≤4, >4)? If you rent agricultural machinery, how much does it cost per mu in 2019 (yuan/mu, single choice: <100, ≥100 & <150, ≥150 & <200, ≥200 & <250, ≥250)?
4. How many people in your family do you need for growing other crops on a yearly basis? How many months does everyone work on average (months, single choice: <2, ≥2 & <4, ≥4 & <6, ≥6 & <8, ≥8)? If you hire workers, how many workers do you need to hire? On average, how many months does each worker work (months, single choice: <2, ≥2 & <4), ≥4 & <6, ≥6 & <8, ≥8)? How much do you pay for the workers (yuan/person·month, single choice: <1000, ≥1000 & <2000, ≥2000 & <3000, ≥3000 & <4000, ≥4000)?
5. Besides land, machinery and labor, how much do other inputs (seeds, fertilizers, pesticides) cost for growing other crops (yuan/mu, single choice: <1000, ≥1000 & <2000, ≥2000 & <3000, ≥3000 & ≤4000, >4000)? Among these inputs, how much chemical fertilizers do you use (kg/mu, single choice: <40, ≥40 & <80, ≥80 & <160, ≥160 & ≤320, >320), and how much do chemical fertilizers cost (yuan/mu, single choice: <100, ≥100 & <200, ≥200 & <400, ≥400 & ≤800, >800)? How much pesticides do you use (kg/mu, single choice: <0.5, ≥0.5 & <1.0, ≥1.0 & <2.0, ≥2.0 & ≤3.0, >3.0), how much do pesticides cost (yuan/mu, single choice: <10, ≥10 & <20, ≥20 & <40, ≥40 & ≤60, >60)?
6. What is the proportion of rice for own consumption in your family (single choice: <5%, ≥5% & <10%, ≥10% & <15%, ≥15% & ≤20%, >20%)? For other crops (the major one), what is the own-consumption proportion (single choice: <5%, ≥5% & < 10%, ≥10% & <15%, ≥15% & ≤20%, >20%)?
7. What is the price for your rice in 2019 (yuan/kg)? What is the price of your other agricultural products (the major one) (yuan/kg)?
8. How much did your family spend per capita in 2019 (ten thousand yuan/person, single choice: <1.0, ≥1.0 & <2.0, ≥2.0 & <2.5, ≥2.5 & ≤3.0, >3.0)? And what is the proportion of your spending on grains and oils, vegetables and fruits and non-staple food (single choice: <10%, 10% -20%, 20% -25%, 25% -30%, >30%)?
9. Besides farming, does your family have any other source(s) of income? If so, what is the proportion of non-farming income for your total income (single choice: <10%, 10% -20%, 20% -40%, 40% -60%, >60%)?
10. How many people are there in your family? How many people work, contributing to the family's financial resources?
11. How many working people in your family have received college education or higher? How many people have high school as their highest education? How many people have junior middle school as their highest education? How many people have primary school as their highest education?
12. Does your family farm take part in any agricultural cooperatives? Have you received any technical training? If not, are there any training needs for the use of agricultural machinery?
13. What is the age of the major labor person in your family and how many years have/has you/he/she been a farmer?
14. Your family's main decision-maker in agricultural production is male or female?
15. Which district do you live in (single choice: Baoshan, Jiading, Minhang, Songjiang, Qingpu, Fengxian, Jinshan, Chongming, PudongNew)?

## Part B. History of Questionnaire Development

The survey questionnaire was designed based on the modeling data needs, and has drawn experience from the 2017 summer field survey of 17 horticulture farms across Shanghai (a project funded by Shanghai Municipal Commission of Agriculture and Rural Affairs) and the 2018 university “summer practice” project about the status of agricultural machinery use and new technology adoption in Shanghai. Co-authors who took a temporary post at or has kept frequent communications with the Shanghai Municipal Commission of Agriculture and Rural Affairs, have provided constructive comments and guidance on revising the form (such as wording) during 2019. Scientists working at the School of Agriculture and Biology at Shanghai Jiao Tong University (SJTU), were also consulted upon for technical contents pertaining to the practices of rice and vegetables production in Shanghai.

Regarding the implementation of the survey, it was electronically done. The user-end survey form (adapted for smartphone and computer) was programmed by Wenjuan.com ([www.wenjuan.com](http://www.wenjuan.com)), a professional survey company that has developed business with both industry and academia, and they have an office in proximity to SJTU’s Xuhui Campus. For the contents of the form, instead of using open-end questions, the survey form provides “choices” (close-ended) only for most of the questions, including the range levels of chemical fertilizers and pesticides applied.
