# Supplementary material for: Operating pesticide use reduction within the boundary of food security in peri-urban settings
Source: Fundam Res. 2022 Apr 18;2(4):635–47. doi: 10.1016/j.fmre.2022.04.003 (PMC11197716; doi:10.1016/j.fmre.2022.04.003)
Supplement: Supplementary file 3 [file mmc3.docx]

## Supplemental File S3

## Additional Figures Showing Full Version Results


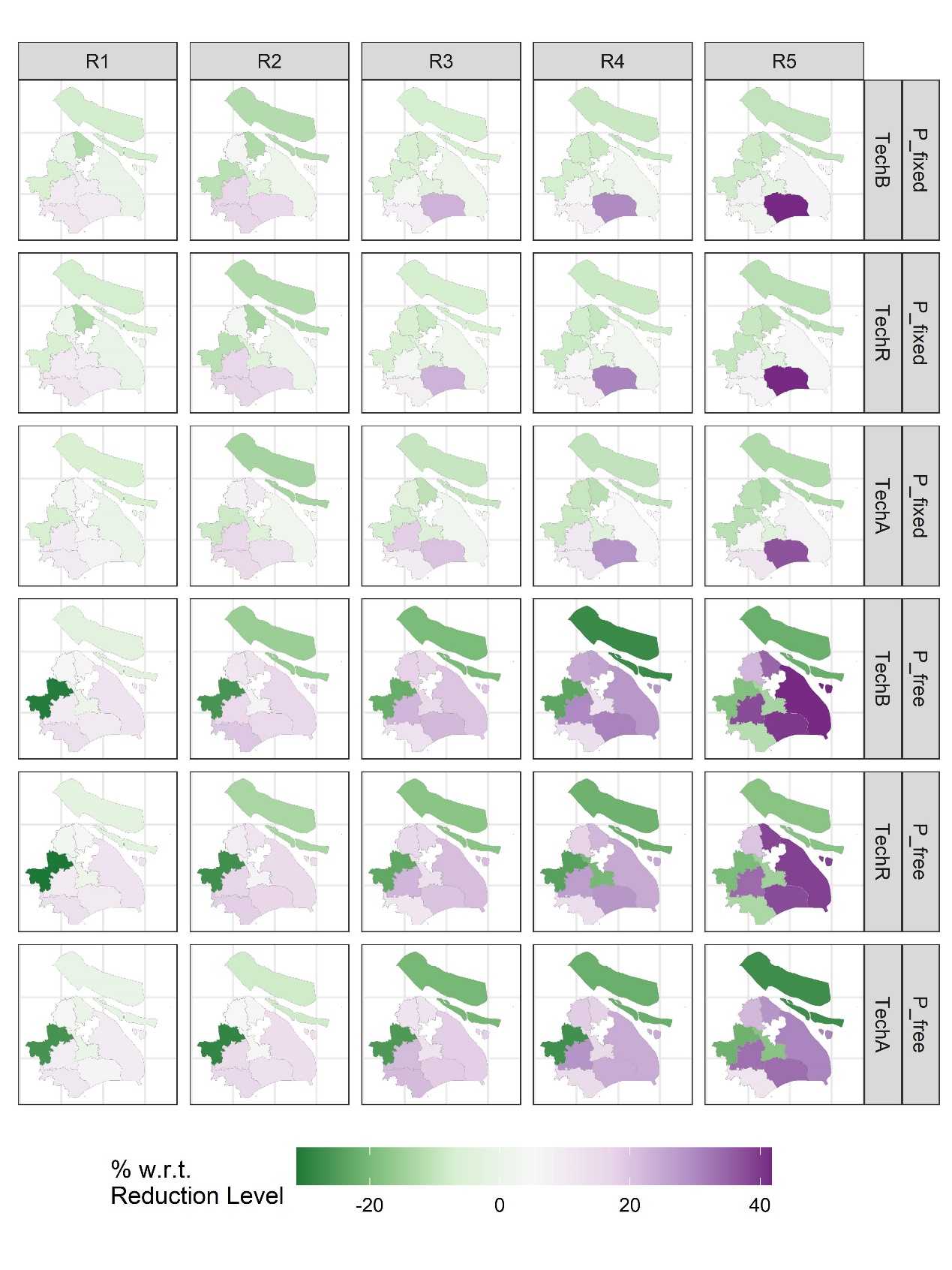


**Fig. S1:** Projected percentage changes in pesticide use levels with respect to assumed district-level usage under different reduction level scenarios (R1-R5), for each district across peri-urban Shanghai under various “Tech + Regionwide imposition” scenarios.


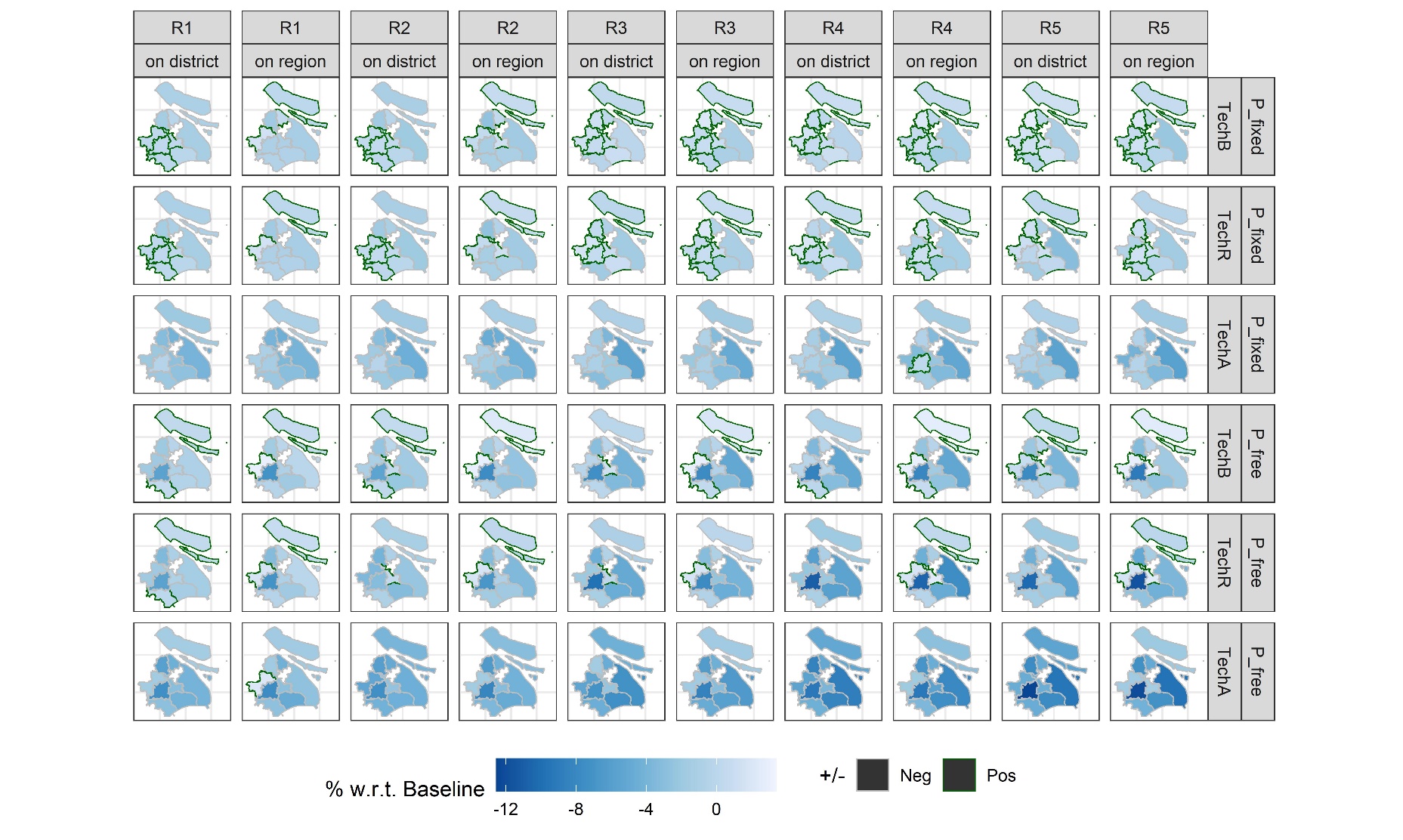


**Fig. S2:** Projected percentage changes in use intensity across districts in Shanghai under various scenarios. Darker colors indicate larger decreases. Districts with borders in dark green are the ones seeing increases in pesticide use intensity.
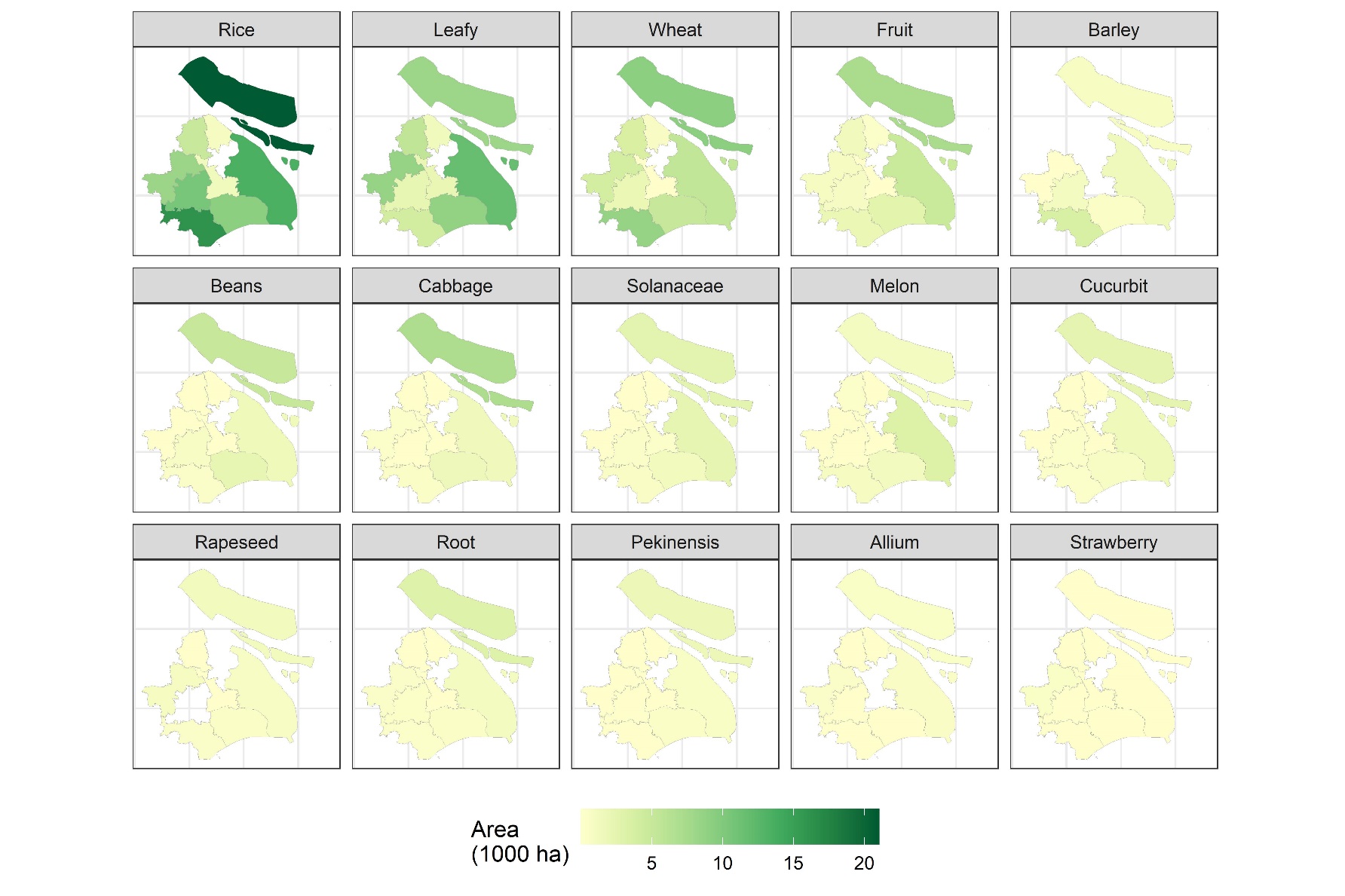


**Fig. S3:** Base (2015) district-level distribution of areas for the fifteen crops modeled in this study.


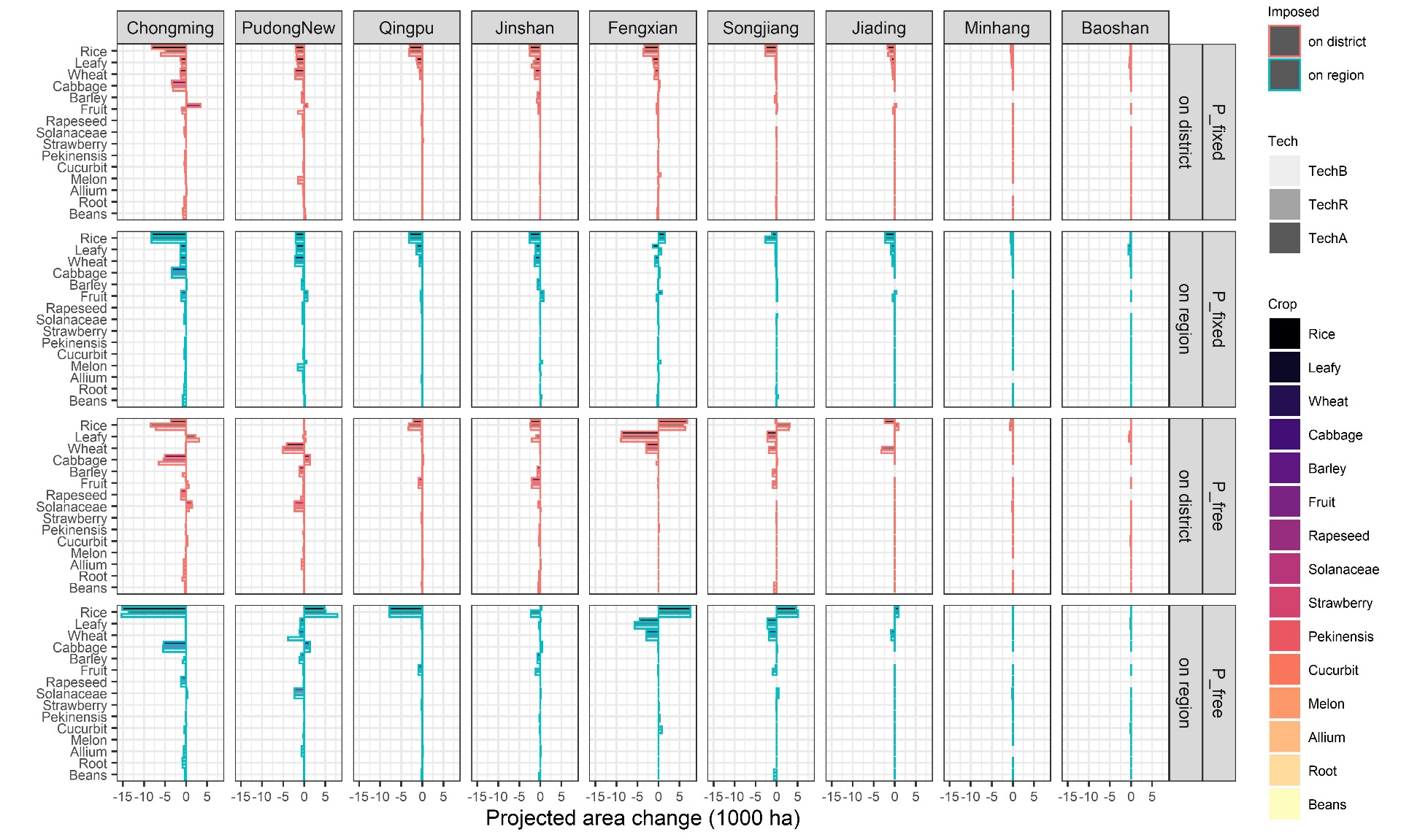


**Fig. S4:** Projected area changes for the fifteen crops by district under various scenarios.


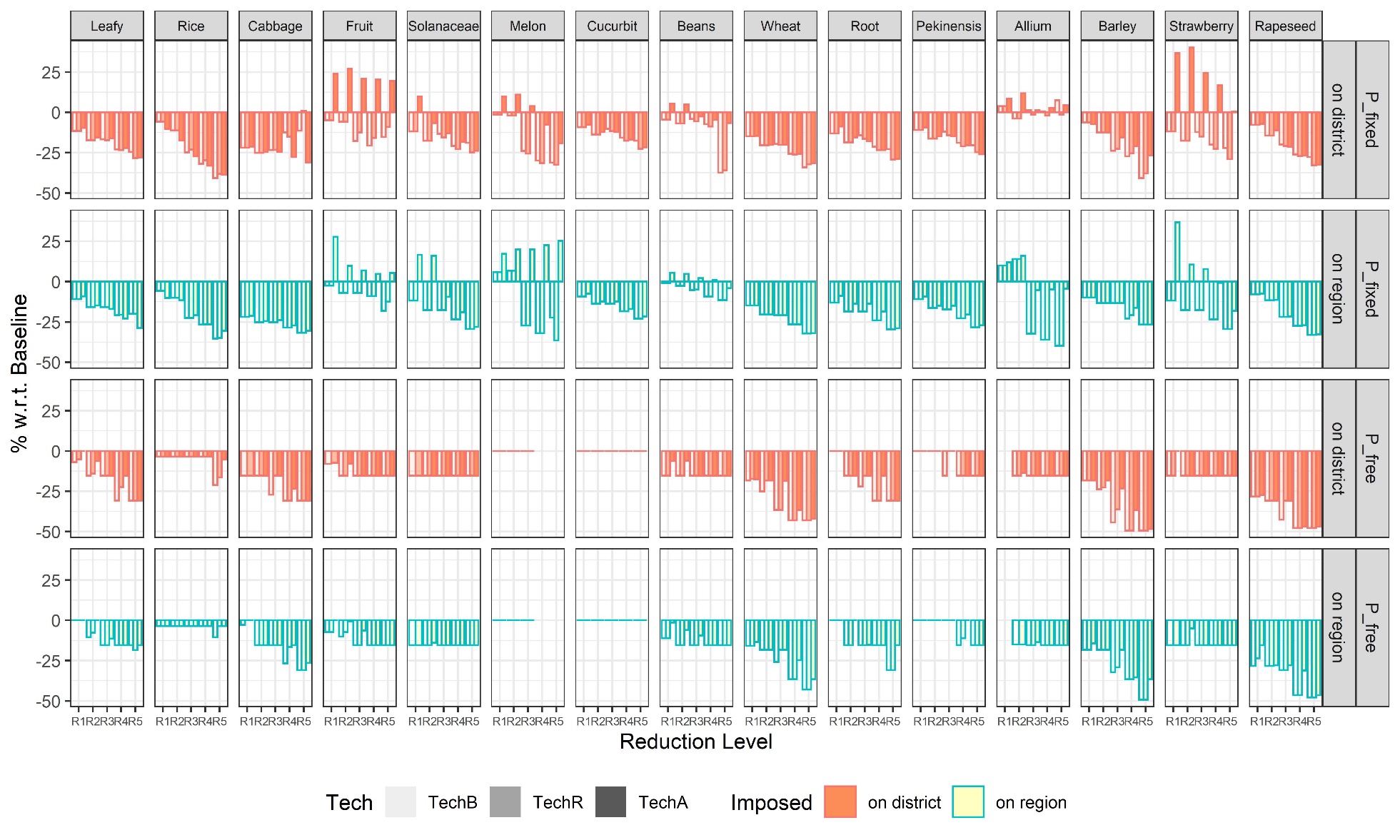


**Fig. S5:** Projected percentage changes in production levels with respect to baseline scenarios by crop under different scenarios.


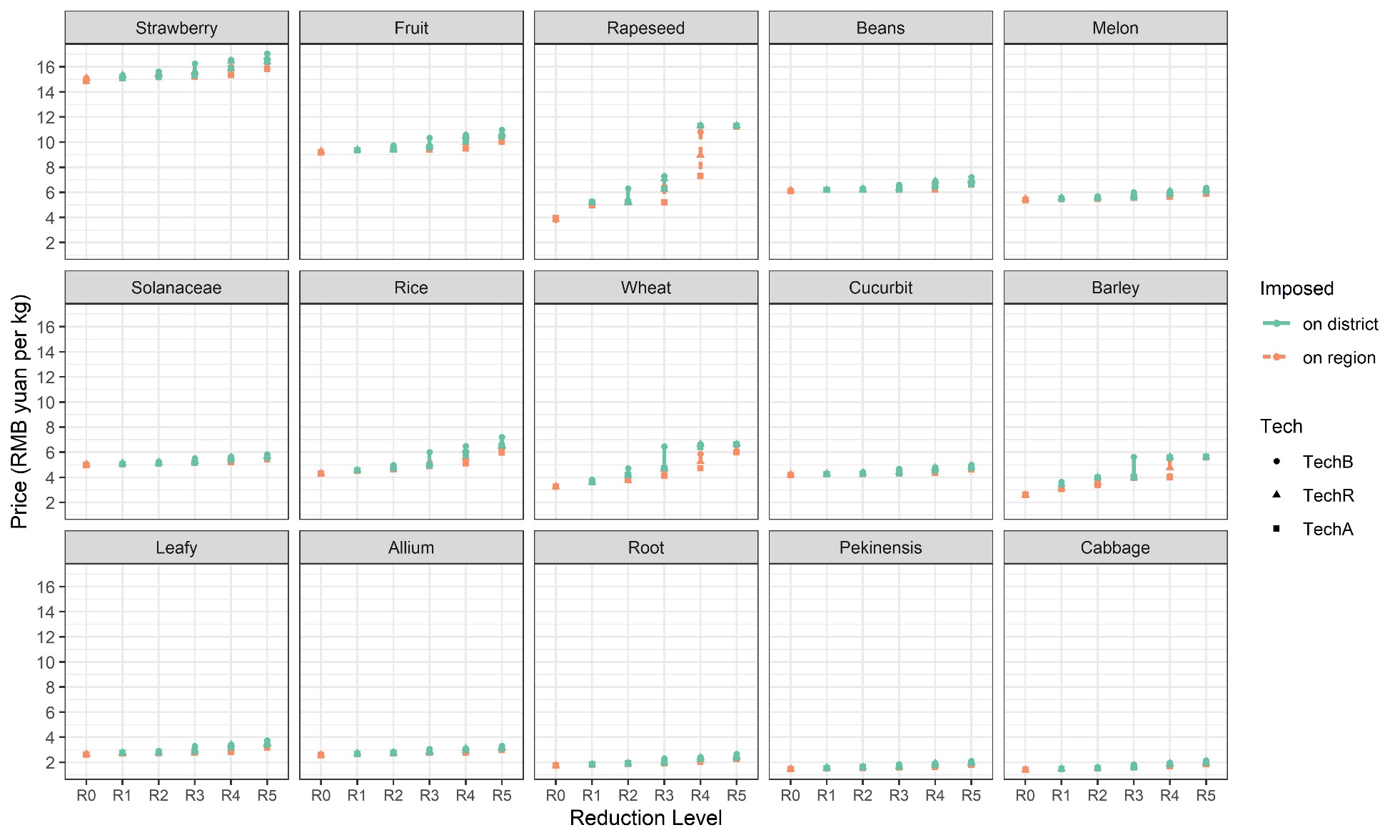


**Fig. S6:** Projected prices by crop under various “P_free” scenarios that assume a closed Shanghai agricultural economy.
